# Syntaxin of plants71 plays essential roles in plant development and stress response via regulating pH homeostasis

**DOI:** 10.3389/fpls.2023.1198353

**Published:** 2023-06-05

**Authors:** Hailong Zhang, Jingwen Zhou, Xiaoyue Kou, Yuqi Liu, Xiaonan Zhao, Guochen Qin, Mingyu Wang, Guangtao Qian, Wen Li, Yongshun Huang, Xiaoting Wang, Zhenjie Zhao, Shuang Li, Xiaoqian Wu, Lixi Jiang, Xianzhong Feng, Jian-Kang Zhu, Lixin Li

**Affiliations:** ^1^ Key Laboratory of Saline-Alkali Vegetation Ecology Restoration, Ministry of Education, College of Life Sciences, Northeast Forestry University, Harbin, China; ^2^ Institute of Crop Science, College of Agriculture and Biotechnology, Zhejiang University, Hangzhou, China; ^3^ Institute of Advanced Agricultural Sciences, Shandong Laboratory of Advanced Agricultural Sciences, Peking University, Weifang, China; ^4^ State Key Laboratory of Tree Genetics and Breeding, Northeast Forestry University, Harbin, China; ^5^ Key Laboratory of Soybean Molecular Design Breeding, Northeast Institute of Geography and Agroecology, Chinese Academy of Sciences, Changchun, China; ^6^ Institute of Advanced Biotechnology and School of Life Sciences, Southern University of Science and Technology, Shenzhen, China; ^7^ Center for Advanced Bioindustry Technologies, Chinese Academy of Agricultural Sciences, Beijing, China

**Keywords:** AtSYP71, pH homeositasis, ROS homeostasis, cell wall biosynthesis and dynamics, root development, vesicle trafficking

## Abstract

SYP71, a plant-specific Qc-SNARE with multiple subcellular localization, is essential for symbiotic nitrogen fixation in nodules in *Lotus*, and is implicated in plant resistance to pathogenesis in rice, wheat and soybean. *Arabidopsis* SYP71 is proposed to participate in multiple membrane fusion steps during secretion. To date, the molecular mechanism underlying SYP71 regulation on plant development remains elusive. In this study, we clarified that AtSYP71 is essential for plant development and stress response, using techniques of cell biology, molecular biology, biochemistry, genetics, and transcriptomics. *AtSYP71*-knockout mutant *atsyp71-1* was lethal at early development stage due to the failure of root elongation and albinism of the leaves. *AtSYP71*-knockdown mutants, *atsyp71-2* and *atsyp71-3*, had short roots, delayed early development, and altered stress response. The cell wall structure and components changed significantly in *atsyp71-2* due to disrupted cell wall biosynthesis and dynamics. Reactive oxygen species homeostasis and pH homeostasis were also collapsed in *atsyp71-2*. All these defects were likely resulted from blocked secretion pathway in the mutants. Strikingly, change of pH value significantly affected ROS homeostasis in *atsyp71-2*, suggesting interconnection between ROS and pH homeostasis. Furthermore, we identified AtSYP71 partners and propose that AtSYP71 forms distinct SNARE complexes to mediate multiple membrane fusion steps in secretory pathway. Our findings suggest that AtSYP71 plays an essential role in plant development and stress response *via* regulating pH homeostasis through secretory pathway.

## Introduction

Cell wall is an important feature of plant cells which provides cell shapes and strength (Keegstra, 2010). Cell wall is a complex extracellular matrix containing cellulose, hemicellulose, polysaccharides, lignin and a small amount of functional glycoproteins (Kumar and Turner, 2015), playing essential roles in cell morphogenesis, signal transduction, development, defense and stress tolerance (Fry, 2004; Liu et al., 2018). Plant cell wall is usually divided into two categories: primary cell wall (PCW) and secondary cell wall (SCW) (Keegstra, 2010). In *Arabidopsis*, the PCW consists of cellulose microfibrils cross-linked by xyloglucan (XyG), and embedded in pectin matrix (Gigli-Bisceglia et al., 2020), and the SCW consists of cellulose, hemicellulose, lignin, and xylans (Meents et al., 2018). XyG is the most enriched hemicellulose in PCWs (Julian and Zabotina, 2022). In addition, cell wall-specific proteins (e.g. extensins, expansins, hydroxyproline-/glycine-rich proteins and glycoproteins), polysaccharides and polyphenols are related to cell wall dynamics and response to environmental stimuli (Zhu, 2018). Lignin plays essential roles on plant growth and development and acts as a physical barrier against biotic and abiotic stress (Liu et al., 2018). Lignin is a heteropolymer of monolignols synthesized primarily through the general phenylpropanoid pathway (GPP), a main synthetic pathway for secondary metabolites e.g. flavonoids etc. (Geng et al., 2020). Lignin precursors are secreted by plasma membrane (PM)-localized transporters such as ATP binding cassette (ABC) transporters (Kaneda et al., 2010; Li and Chapple, 2010; Miao and Liu, 2010; Alejandro et al., 2012), and diffuse in both PCWs and SCWs for active lignification (Tobimatsu et al., 2013; Schuetz et al., 2014; Pandey et al., 2016; Geng et al., 2020). The monolignols polymerization is catalyzed by plant-specific class III peroxidases (PRXs) and laccases (Dixon and Barros, 2019). PRXs, the secretory peroxidases accumulated in the plant cell wall or the vacuole(Kidwai et al., 2020), are related to cell wall polymerization such as lignification, suberisation and cross-linking of cell-wall constituents, and consumption of hydrogen peroxide (H_2_O_2_), etc. (Meng et al., 2021). PRXs reduce H_2_O_2_ to oxidize monolignols (Meyer et al., 2009; Didi et al., 2015). Plant laccases, the multicopper oxidases, are necessary and nonredundant with PRXs for lignification during vascular development in *Arabidopsis*. Laccase 4 (LAC4)/11/17 are redundantly required for asymmetric lignification (Zhao et al., 2013). Unlike PRXs, laccases reduce O_2_ to H_2_O to oxidize monolignols without consuming reactive oxygen species (ROS) (Wang et al., 2015).

Flavonoids are broadly distributed in plants, and is essential for plant development and defense (Falcone Ferreyra et al., 2012; Sharma et al., 2019). In plant cells, flavonoids are mainly synthesized in the cytoplasmic face of the endoplasmic reticulum (ER) and then uploaded into the ER lumen (Zhao and Dixon, 2010; Falcone Ferreyra et al., 2012; Zhao, 2015). The PM-localized flavonoid transporters are mainly ABC transporters (Ferrer et al., 2008; McFarlane et al., 2010; Sharma et al., 2019), e.g. ABCG (ATP binding cassette sub-family G) protein. ABCG10 secrets isoflavonoids to the apoplast for plant defense (Klink et al., 2017), ABCG1 and ABCG16 are required for secretion of suberin and pollen wall synthetic materials (Zhao, 2015; Shanmugarajah et al., 2019; Liu et al., 2020; Dhara and Raichaudhuri, 2021). SNARE-mediated exocytosis pathways or nondirectional secretion are also proposed to mediate in the efflux of flavonoids into the extracellular space (Zhao and Dixon, 2010) Higher plants have developed a unique vesicle trafficking system to meet the needs of plant development and environmental adaption. Vesicle trafficking involves multiple organelles, and carries out material communication and signal transmission (Adnan et al., 2019). The Golgi functions as a vesicle transport hub, accepting ER-derived vesicles, and facilitating sorting of the cargo into various vesicles. Moreover, the Golgi is also a factory which holds protein post-translational modification (e.g. glycosylation) and substance synthesis (e.g. hemicellulose biosynthesis and assembly) (Bui et al., 2021). The protein glycosylation initiates in the ER lumen and is completed in the Golgi cisternae (Frankova and Fry, 2013). For example, glycosylation of arabinogalactan proteins (AGPs) is completed by Glycosyltransferases (GTs) at the Golgi (Frappaolo et al., 2020).The trans-Golgi network (TGN) is a sorting hub for both exocytosis and endocytosis (Heinze et al., 2020). At the TGN, hemicelluloses and secretory proteins are sorted into endosomes and targeted to the PM (Kang et al., 2011). The TGN-PM cycling pathway and vacuolar degradation pathways coordinately fine-tune homeostasis of the PM proteins, especially under environmental stimuli (Meents et al., 2018; Gigli-Bisceglia et al., 2020). Thus it can be seen that Golgi apparatus plays an important role in the secretion of cell wall biosynthetic materials and in stress response. The process of vesicle trafficking includes budding, directional movement, tethering, anchoring and membrane fusion (Cui et al., 2022), and each step is regulated by various factors, such as coatomer, SM, tether, SNARE and Rab proteins, which are highly conserved in yeast, mammals and plants (Singh and Jurgens, 2018). The soluble N-ethylmaleimide-sensitive factor attachment protein receptor (SNARE) proteins play an essential role on membrane fusion of arriving vesicles with the target membrane (Yoon and Munson, 2018; MartiniEre and Moreau, 2020). SNAREs usually contain an N-terminal region, a SNARE motif and a transmembrane domain (Fasshauer and Margittai, 2004). According to the conserved amino acids, glutamine or arginine residues in core of SNARE domain, SNARE proteins are divided into Q-SNAREs on the target membrane and R-SNAREs on the vesicle (Fasshauer et al., 1998). Q-SNAREs include Qa-, Qb- and Qc-SNARE, the three Q-SNAREs combine with R-SNARE to form trans-SNARE complex facilitating membrane fusion (Antonin et al., 2000). SYP71 is a plant-specific Qc-SNARE (Sanderfoot et al., 2000), with multiple subcellular localization of the ER, plasma membrane (Suwastika et al., 2008), endosome and cell plate (Bao et al., 2012; Klink et al., 2017). It is reported that SYP71 is essential for symbiotic nitrogen fixation in nodules in *Lotus* (Hakoyama et al., 2012), and participates in pathogen resistance in *Glycine max* (Klink et al., 2017), rice (Bao et al., 2012), wheat (Liu et al., 2016) and *Arabidopsis* (Wei et al., 2013). *Arabidopsis* SYP71 is proposed to localize at the PM, endosome and ER, and be involved in multiple membrane fusion steps during secretion (Tyrrell et al., 2007). Moreover, AtSYP71 coordinates with Qa-SNARE KNOLLE, Qb-SNARE NPSN11 and R-SNAREs VAMP721/722 to facilitate cell plate formation during cytokinesis (Suwastika et al., 2008; Bao et al., 2012; Yoon and Munson, 2018). However, the molecular mechanism underlying AtSYP71 regulatory roles on plant development is elusive.

In this study, we illustrate that AtSYP71 is essential for plant development and stress response. Homozygous mutant *atsyp71-1* was seedling lethal, the knockdown mutants *atsyp71-2* and *atsyp71-3* exhibited severe early development defects, such as short roots and delayed bolting. Transcriptome analysis indicated that cell wall biosynthesis, metabolism and stress response were greatly affected in *atsyp71-2.* In response to transcriptomic data, the cell wall components and structures significantly altered, and ROS and pH homeostasis were disrupted in *atsyp71-2*. Furthermore, secretion was blocked in *atsyp71-2*. AtSYP71-interacting SNAREs were identified by pull down-LC-MS/MS analysis. The results suggest that AtSYP71 forms distinct SNARE complex at multiple steps in secretory pathway. Our findings suggest that AtSYP71 regulates pH homeostasis by controlling vesicle transport pathways, thereby affecting plant development and stress responses.

## Materials and methods

### Plant materials and growth conditions

*Arabidopsis thaliana* ecotype Col-0 was used as wild type. T-DNA mutants were generated from Col-0. *atsyp71-1* (GABI_367A08), *atsyp71-2* (SALK_081547), *atsyp71-3* (SALK_201897) and *atsyp71-4* (SAIL_813_A05) were obtained from the *Arabidopsis* Biological Resource Center (ABRC) at Ohio State University (https://abrc.osu.edu). Homozygous plants were isolated by PCR genotyping using the insertion-specific primers listed in **Supplementary Table 3**. The seeds were surface-sterilized and sown either in soil or on 1/2 Murashige and Skoog medium (PhytoTech) with 1% (w/v) sucrose and 0.8 or 1.2% agar. Plants were grown at 22°C under 16 h: 8 h/light: dark cycles, horizontally or vertically as needed. The root length was measures using ImageJ software.

Transgenic plants (Col-0) expressing myc (TAP)-tagged *AtSYP71* were generated using a modified pNTAPa vector described by (Li et al., 2013).

### Antibody preparation

To prepare antibodies against AtSYP71, a polypeptide (Cys-LPARIEAIPDGTA GGPKSTSAWTPSSTTSRPDIKFDSDGRFDDDYFQESN) was synthesized and conjugated to a Carrier protein KLH linked by an N-terminal Cys residue. The AtSYP71 peptide–KLH conjugates were injected into two rabbits to generate antibodies. The antibodies were subjected to ProteinA/G purification from the serum and ELISA detection. Polypeptide synthesis and antibody preparation were commissioned to GL Biochem (Shanghai) Ltd.

### Immunoblotting

Immunoblot analysis was performed as described previously (Li et al., 2006). Antibodies were diluted as follows: anti-AtSYP71, 1:2,000. The secondary antibody was against rabbit IgG (ZB2301 and ZSGB-BIO), 1:5000. Immunoreactive signals were detected using a chemiluminescence detection system (LAS-4000 and FYJIFILM).

### Confocal microscopy

Fluorescent images were obtained using a point scanning confocal microscope (Leica TCS SP8). Confocal imaging was preset for GFP (Ex:488 nm, Em:500–550 nm) or for propidium iodide staining (Ex:543 nm, Em: 580–640 nm).

### Yeast two-hybrid Assay

For the yeast two-hybrid assay, the fragments of *AtSYP71* (cytosolic region), *MIP2* (Sec39 domain) and *MIP3* were amplified using cDNA obtained from seedlings with specific primers and ligated into pEASY-Blunt vector (TransGen, #CB101-01), respectively. After Sanger sequencing confirmation, the fragments were transferred into *pGADT7* or *pGBKT7* vectors, respectively. *AtSYP81*, *AtSEC20* and *MAG2* constructs were generated in our previous study (Li et al., 2006; Zhao et al., 2018). The paired constructs were introduced into *Saccharomyces cerevisiae* strain AH109 (Clontech) and selected on SD/-Leu/-Trp medium. The interactions were detected on SD/-Leu/-Trp/-His/-Ade medium.

### RNA extraction and RT-qPCR

Total RNA was extracted as described previously (Guan et al., 2021) using 9-day-old seedlings grown on 1/2MS medium horizontally. Reverse transcription Quantitative polymerase chain reaction (RT-qPCR) was performed according to the manufacturer’s instructions. The specific primers are listed in **Supplementary Table 3**.

### RNA sequencing

Total RNA was extracted from 9-day-old seedlings grown on 1/2MS medium horizontally. Illumina cDNA libraries were constructed with the TruSeq RNA Sample Prep Kits v2 (Illumina, San Diego, CA, USA) following the manufacturer’s protocol. Sequencing of the cDNA libraries was performed by pair-end methousing an Illumina HISEQ-x10 with a 150-bp read length and a sequence depth ~20 million uniquely mapped reads. Three biological replicates per sample. The data presented in the study are deposited in the NCBI repository, the BioProject ID is PRJNA971388.

### Sequence trimming, mapping and expression level determination

Reads were trimmed using the CLC Genomics Workbench 6.5.1 (CLC bio, Denmark) with the following parameters: ‘quality scores-0.005; trim ambiguous nucleotides-2; remove 5’ terminal nucleotides-1; remove 3’ terminal nucleotides-1; discard reads below length 25’. Trimmed reads were mapped using the RNA-seq mapping algorithm implemented in the CLC Genomics Workbench to the reference *Arabidopsis thaliana* genome (TAIR10) allowing only unique mapping (length fraction=1, similarity fraction=0.95). In order to estimate the influence of non-uniquely mapped reads on gene expression we also mapped reads using the same software and parameters as indicated above, but allowing multiple mapping (up to 10 hits). For each gene, total gene reads (TGR) was determined as the sum of all reads mapped to this gene. To avoid bias due to different library sizes, TGR values were normalized by a size factor as described in Anders and Huber (Anders and Huber, 2010).

### Identification of DEGs

DEGs were identified using the R package ‘DESeq’ (Anders and Huber, 2010). A false discovery rate (FDR) of 0.05 and a fold change of 2.0 were chosen as the threshold for significantly differential expression. The original transcriptomic data of *atsyp71-2* vs Col-0 is in **Supplementary Table 1**.

### GO enrichment analysis

Downregulated and upregulated DEGswere analyzed for GO and other annotation (as key words or protein domain) enrichment using the DAVID gene functional annotation tool https://david.ncifcrf.gov/, with an FDR value of 0.05 and a fold change of category representation of 2.0 as the threshold of significance (Huang da et al., 2009a; Huang da et al., 2009b).

### Enrichment analysis of differential gene KEGG

KEGG enrichment analysis was performed using KOBAS (v2.0). Rich factor, *P-*value and the number of genes indicated the degree of KEGG enrichment. Rich factor means the ratio of enriched DEGs to all genes annotated in the pathway. Therefore, the higher the Rich factor value, the greater the enrichment of the pathway. Parameter setting: Corrected *P*-Value < 0.05.

### Determination of lignin

The first segment of stems from ten-weeks-old plants were harvested and dried at 80°C to constant weight, then were ground and passed through a 40 mesh sieve. About 5 mg was put into a 10 mL glass test tube with 1 mL of glacial acetic acid containing 30% acetyl bromide and 40 uL of perchloric acid, sealed with a sealing film, fully mixed, then incubated in water bath at 80°C for 40 min, shaking every 10 min. After natural cooling, 1 mL of 2 mol/L NaOH and glacial acetic acid (equal volume) were added and fully mixed. Add 1,960 uL of acetic acid to 40 uL of supernatant and fully mixed. Then, absorbance of the mixture was determined by spectrophotometer at 280 nm wavelength versus the prepared blank.

### Determination of cellulose

The first segment of stems from ten-weeks-old plants were harvested and incubated overnight in 80% ethanol at 65°C. Dry materials were ball milled to fine powder. Cellulose content was determined by Anthrone sulfuric acid colorimetry using Cellulose determination kit (CLL-2-Y, Comin, Suzhou Keming Biotechnology Co., Ltd.) according to Xiao et al. (2016). D-Glc (Sigma) was used as a standard for calculation of cellulose content. Three repeats per sample.

### Determination of total flavonoids

The flavonoid content was determined by colorimetric assay (Kim et al., 2003). A 250 μL of standard solution of rutin at different concentrations or appropriately diluted samples were added to 10 ml volumetric flask containing 1 ml of distillate water, respectively, then, 75 μl of NaNO_2_ (5%) was added and mixed thoroughly. After 6 min of incubation, 75 μl of AlCl_3_ (10%) was added, fully mixed and incubated for 6 min, then 500 μl of NaOH (1N) was added. Immediately, the solution was diluted by adding 2.5 ml of methanol and mixed thoroughly. Absorbance of the mixture was determined by spectrophotometer at 506 nm wavelength versus the prepared blank. Total flavonoid compounds in plant were indicated as mg rutin equivalents (CE mg/ml). Three repeats per sample.

### Analysis of enzyme activities

For determination of activities of ROS-scavenging enzyme, 0.2 g of roots from nine-day-old seedlings were ground in 1.6 ml of precooled 50 mM phosphate-buffered saline (PBS) buffer (pH7.8). The homogenate was centrifuged at 16,000 g for 20 min at 4°C, and the supernatant was used for the assays. Determination of activities of peroxidase (POD), superoxide dismutase (SOD) and superoxide catalase (CAT) were performed according to established protocols (Luo et al., 2020). Three biological replicates per sample.

### Chemical determination of cell wall monosaccharides

0.1g dry powder of the first stem segments from ten-weeks-old Col-0 and *atsyp71-2* plants were used for determination of cell wall monosaccharides according to the method reported by Liu et al. (Liu et al., 2022a).

### Histological analysis and immunofluorescence analysis

The first stem segments from 10-weeks-old Col-0 and *atsyp71-2* plants were fixed and made into paraffin slices (Wang et al., 2020), and subjected to immunofluorescence analysis according to previously reported method (Wang et al., 2016).

### Pull down assay and Shotgun LC-MS/Ms analysis

Pull-down assay was performed as described previously using a μMACS™ epitope tag protein isolation kit (Anti-c-myc MicroBeads, Miltenyi Biotec, Order No. 130-091-123). Two grams of ten-day-old seedlings were used for initiation. The eluates were used for Shotgun liquid chromatography-tandem mass spectrometry (LC-MS/MS) analysis.

LC-MS/MS analysis was mainly performed as described previously (Qin et al., 2017), with slight modifications.

## Results

### AtSYP71 is essential for plant growth and development

**Figure 1:**
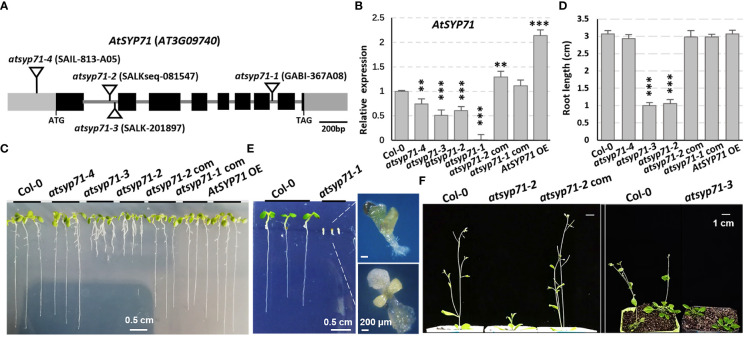
AtS*YP71* is essential for plant morphogenesis and early development. **(A)** AtSYP71 gene structure diagram. Black boxes represent exons, gray lines represent introns, and gray boxes represent untranslated regions (UTRs). The triangles indicate the T-DNA insertion sites of the *atsyp71* mutants. **(B)** Statistics of RT-qPCR detection of the relative expression levels of *AtSYP71* in *atsyp71* mutants, the *atsyp71* complementation lines and *AtSYP71* overexpression line. Three independent experiments per sample, two repeats per experiment. **(C)** Phenotype of seven-day-old seedlings of Col-0, *atsyp71* mutants, *atsyp71-2* com and *AtSYP71* OE lines. **(D)** Statistics of primary root length of the displayed genotypes (n ≥ 44). Three biological replicates per sample. **P < 0.01; ***P < 0.001. Student’s t test. **(E)** Five-day-old seedlings of Col-0 and *atsyp71-4* mutant. Magnified pictures highlighted growth defects of *atsyp71-4*. Bar, 0.5 cm. **(F)** Bolting of *atsyp71-2* and *atsyp71-3* was delayed. Four- (right panel) and five (left panel)-week-old plants of the displayed genotypes.

To explore the biological function of AtSYP71 on plant growth and development, four T-DNA insertion mutants of *AtSYP71*, *atsyp71-1* to *atsyp71-4* were obtained. Genotyping and sequencing confirmed that T-DNA was inserted in 5’-UTR, different sites of the first intron region and the seventh intron of *AtSYP71*, respectively, in *atsyp71-1* to *atsyp71-4* (**Figure 1A**). RT-qPCR analysis indicated that *AtSYP71* expression was eliminated in *atsyp71-1*, and was significantly downregulated in *atsyp71-2* to *atsyp71-4* (**Figure 1B**). Consequently, *atsyp71-2* to *atsyp71-4* were knockdown alleles, and *atsyp71-1* was knockout allele. The complementation lines of *atsyp71-1* (*atsyp71-1* com) and *atsyp71-2* (*atsyp71-2* com) were generated by crossing *atsyp71-1* (using heterozygous plant) and *atsyp71-2* with *pAtSYP71*::*GFP-AtSYP71*-expressing wild type (Col-0) line, respectively. The complemented lines restored the *AtSYP71* expression in *atsyp71-1* and *atsyp71-2* mutants (**Figure 1B**). *p35S::TAP-AtSYP71* (*AtSYP71* OE) conferred significantly increased *AtSYP71* expression in wild-type background (**Figure 1B**). Comparison of the early development phenotypes of Col-0, *atsyp71* mutants, complementation lines (*atsyp71-1/-2* com) and *AtSYP71* OE revealed that seven-day-old seedlings of *atsyp71-2* and *atsyp71-3* developed much shorter primary roots (about 67% decrease) and smaller shoots than Col-0, while, the root length and shoots of *atsyp71-4* and *AtSYP71* OE had no significance compared with that of Col-0 (**Figures 1C, D**). However, the development of the knockout allele *atsyp71-1* was inhibited. After germination, roots of *atsyp71-1* cannot elongate, cotyledons were albino or vitrified, and the true leaves were very small and turned yellow and vitrified (**Figure 1E**). And the so-called seedlings dead at about ten days after germination. The bolting of *atsyp71-2* and *atsyp71-3* plants was significantly delayed compared with that of Col-0 (**Figure 1F**). The phenotypes of *atsyp71-1* com and *atsyp71-2* com were restored (**Figures 1C, D, F**). These results suggest that AtSYP71 is essential for plant morphogenesis and early development.

### Transcriptome analysis of *atsyp71-2* mutant

**Figure 2:**
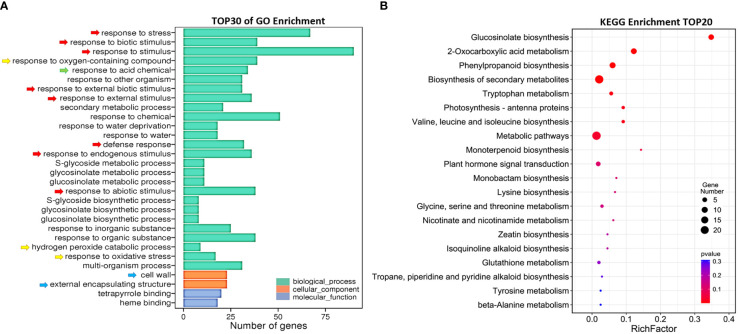
Comparative transcriptome analysis of DEGs in *atsyp71-2*. **(A)** Gene ontology (GO) enrichment analysis of DEGs. X axis represents DEG number. Y axis represents GO terms. **(B)** Top 20 pathways of KEGG enrichment analysis. X axis represents enrichment factors; Y axis represents pathways. The color bar indicates the *P* value, the circle size indicates DEG number.

To explore the mechanism of *AtSYP71* regulatory role on plant growth and development, we performed transcriptome analysis using roots of nine-day-old Col-0 and *atsyp71-2* seedlings. The sequencing obtained 34,524 and 33,113 reliable clean reads above 100 bp in Col-0 and *atsyp71-2*, respectively. After comparison using tophat2 software, 87.68% and 86.59% of clean reads matched to the reference genome sequence, respectively. Statistical evaluation of sequencing quality value showed that base Q30% was 92.37% and 92.16%, respectively (**Supplementary Figure 1A**), indicating the high quality of transcriptome sequencing and reliable original data for subsequent analysis. Volcano map indicated the overall distribution of the differentially expressed genes (DEGs). A total of 165 DEGs were identified, of which 69 were up regulated, and 96 were down regulated (**Supplementary Figure 1B**). Gene Ontology (GO) analysis revealed that in Biological process (BP), the DEGs were enriched in Response to biotic and abiotic stimuli/stress, Defense responses (**Figure 2A**, red arrows), and Redox processes (yellow arrows), etc. In Cellular component (CC), DEGs were enriched in Cell wall and External encapsulating structure (blue arrows). Kyoto Encyclopedia of Genes and Genomes (KEGG) enrichment analysis indicates that DEGs were mainly enriched in Glucosinolate Biosynthesis, 2-Oxocarboxylic acid metabolism and Phenylpropanoid biosynthesis, Metabolic and Secondary metabolic processes, and Amino acids metabolism, etc (**Figure 2B**). These results suggest that down regulation of *AtSYP71* disturbed plant response to stimuli and stress through redox processes and metabolism etc.

### The expression of cell wall-relating genes were affected significantly in *atsyp71-2*

**Figure 3:**
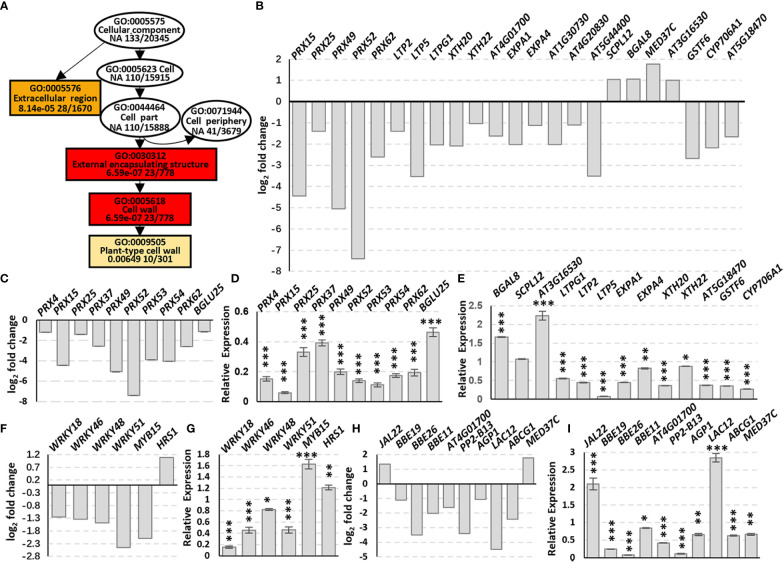
Changes in DEG Enrichment in *atsyp71-2* mutant. **(A)** The enriched GO terms in Cellular component (GO:0005575) of DEGs in Col-0 and *atsyp71-2* seedlings. Rectangles indicate the significant terms. The colors of rectangles and ovals represent the relative significances, ranging from red (the most significant, *P* < 0.0001), orange (the second significant, *P* < 0.001), light yellow (the third significant; *P* < 0.05) and white (no significance). **(B)** Transcriptomic analysis of DEGs in CC. **(C)** Statistics of RT-qPCR analysis of DEG expression levels in **(B)**. **(D, E)** Transcriptomic analysis and statistics of RT-qPCR analysis of DEGs in Phenylpropanoid biosynthesis pathway. **(F, G)** Transcriptomic analysis and statistics of RT-qPCR analysis of some other DEGs related to cell wall biosynthesis. **(H, I)** Transcriptomic analysis and statistics of RT-qPCR detection of Transcription factors related to cell wall biosynthesis. Total RNA for RT-qPCR analysis were from roots of seven-day-old seedlings. All RT-qPCR Data are presented from three independent experiments performed with four technical replicates per sample. *P < 0.05; **P < 0.01; ***P < 0.001; Student’s t-test. Abbreviations: ABCG1, ATP binding cassette sub-family G 1; AGP1, Arabinogalactan protein 1; BBE19/OGOX1, FAD-binding Berberine Bridge Enzyme 19/oligogalacturonide oxidase 1; BGAL8, beta-galactosidase 8; BGLU25, beta glucosidase 25; CYP706A1, cytochrome p450 family 706, subfamily A, polypeptide 1; EXPA1, expansin A1; GSTF6, glutathione s-transferase 6; JAL22, jacalin-related lectin 22; LAC12, LACCASE 12; LTP2 (lipid transfer protein 2); LTPG1 (glycosylphosphatidylinositol-anchored lipid protein transfer 1), PP2-B13, PHLOEM PROTEIN 2-B13; PRX, peroxidase; SCPL12, serine carboxypeptidase-like 12; XTH20, xyloglucan endotransglucosylase/hydrolase 20.

Go enrichment analysis of the DEGs in Cellular component (GO:0005575, CC) indicated that the DEGs were enriched in the Extracellular region (GO:0005576) and External encapsulating structure (GO:0030312) containing Cell wall (GO:0005618) which includes Plant-type cell wall (GO:0009505) (**Figure 3A**, rectangles). The DEGs include five *PRXs* (class III peroxidases), three *LTPs* (lipid transfer proteins), three *XTHs* (xyloglucan endotransglucosylase/hydrolase), five *EXPAs* (expansins) and some other cell wall biosynthesis-related genes, and most of these genes were down-regulated (**Figure 3B**; **Supplementary Table 2**), suggesting that AtSYP71 is related to cell wall biosynthesis and dynamics.

Phenylpropanoid biosynthesis pathway is reported to be involved in cell wall biosynthesis (Lewis et al., 1987; Merali et al., 2007), and the enriched DEGs include nine *PRXs* and *BGLU25*, a β-D-glucosidase gene (**Figure 3C**). The five *PRXs* in CC were also included in this pathway. RT-qPCR analysis indicate that the changes of the DEGs in this pathway and CC were the same, and most of the DEGs had significances (**Figures 3D, E**). Among these factors, PRXs are involved in lignification, suberization, cross-linking of extensins, metabolism of reactive oxygen species (ROS), as well as cell wall dynamics, e.g. cell wall loosening and strengthening, etc. (Almagro et al., 2009; Francoz et al., 2015). XTH enzymes play a role in cell wall loosening through the modification of xyloglucan chains (Opazo et al., 2017). EXPAs are located in the cell wall and activated by low apoplastic pH (Cosgrove, 2005), and act on cell wall loosening resulting in cell elongation (Pacifici et al., 2018). Moreover, BGAL8 (beta-galactosidase) (Chandrasekar and van der Hoorn, 2016), LTPs (lipid transfer proteins) (Chae et al., 2009; Bard et al., 2016), BBE19/OGOX1 (FAD-binding Berberine Bridge Enzyme 19/oligogalacturonide oxidase 1) (Benedetti et al., 2018), ABCG1 (Shanmugarajah et al., 2019) and ATPP2 (Phloem protein 2)-B13 (Bobbili et al., 2018) participate in cell wall biosynthesis and dynamics, and GSTF6 (glutathione s-transferase 6), CYP706A1 (cytochrome p450 family 706, subfamily A, polypeptide 1), BGLU25 (beta glucosidase 25) and SCPL12 (serine carboxypeptidase-like 12) are involved in biosynthesis of flavonoids which is related to stress response (Xu et al., 2004; Su et al., 2011).

In addition to the DEGs in CC, expression of some transcription factors (TFs) related to cell wall dynamics/biosynthesis and stress response also altered significantly (**Figure 3F**). RT-qPCR analysis confirmed that except for *MYB15*, the changes of the TF genes were consistent with those of transcriptomic data, and most of them had significances (**Figure 3G**). It has been demonstrated that MYB15 plays a central role on pathogen-induced lignification (Kim et al., 2020a), and WRKY18 and WRKY53 coordinate with histone acetyltransferase1 (HAC1) to regulate responses to sugars, the structural components of cell wall (Rolland and Sheen, 2005; Chen et al., 2019). WRKY46, WRKY48 and WRKY51 are responsible for abiotic stress response, and HRS1 is involved in nitrogen saturation signaling (Xing et al., 2008; Gao et al., 2011; Chen et al., 2017; Li et al., 2021). To further confirm that the alteration of expression of these genes was resulted from knockdown of *AtSYP71*, we checked the expression of some of the genes randomly in *atsyp71-3*. The results showed the same trends of change (**Supplementary Figure 2**). The significant alteration of expression of these genes in *atsyp71* mutants suggest that AtSYP71 regulates cell wall biosynthesis, dynamics and plant stress response.

### The cell wall components and structure were affected in *atsyp71-2* mutant

**Figure 4:**
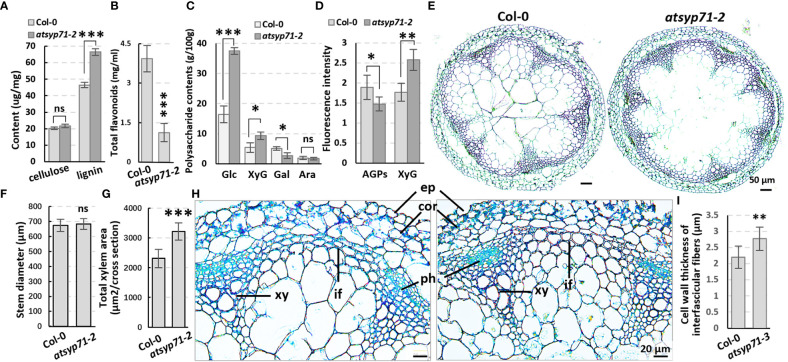
The cell wall components and structure altered in *atsyp71-2* mutant. **(A-C)** Statistics of contents of lignin, cellulose **(A)**, polysaccharides **(B)** and total flavonoids **(C)** in the first section of stems from ten-week-old plants. Three biological replicates per sample. **(D)** Statistics of fluorescence intensity of Immunolabeling of AGPs with anti-LM2 antibody and XXXG xyloglucan with anti-LM15 antibody, respectively.n_Col-0 = _6, n*_atsyp71-2 = _* 7. **(E)** The paraffin sections with Safranine and Fast Green double staining of stems from the same batch as those in **(A)**. **(F, G)** Statistics of stem diameter **(E)** and total xylem area **(F)** shown representatively in **(D)**. n≥8 stems. **(H)** Magnified images of the stem cross sections. **(I)** Statistics of cell wall thickness of interfascicular fiber cells. n≥50 cells. *P < 0.05; **P < 0.01; ***P < 0.001. Student’s t test. Ara, arabinose; Gal, galactose; Glu, glucose; XyG, xyloglucan; co, cortex; ep, epidermis; if, interfascicular fibers; ph, phloem; xy, xylem. ns, no significance.

Transcriptome analysis reveals that in CC, all DEGs were enriched in Cell wall and External encapsulating structure (**Figure 2A**, blue arrows), indicating AtSYP71 is closely related to cell wall homeostasis. Therefore, we first determined the contents of cell wall components in *atsyp71-2* stems. The results indicate that the content of lignin in *atsyp71-2* stems increased significantly, while, the content of cellulose didn’t change obviously (**Figure 4A**), but the content of flavonoids reduced significantly compared with that in Col-0 (**Figure 4B**). Determination of cell wall polysaccharides indicate that the contents of glucose and XyG increased significantly, of galactose decreased significantly, and of arabinose didn’t change significantly compared with that in Col-0 (**Figure 4C**). Immunofluorescence images indicate that the content of AGPs decreased significantly, and of XyG increased significantly which is consistent with the result of polysaccharides determination (**Figure 4D**; **Supplementary Figure 3**). To investigate the effects of changes in the component abundance on cell wall structure, we observed the cross section of stems. The images revealed that the stem diameter didn’t change obviously (**Figures 4E, F**), but the average xylem number and total area in *atsyp71-2* increased significantly compared with that in Col-0 (**Figures 4E, G**). Moreover, the cell wall thickness of interfascicular fiber (IF) cells was significantly thicker than that of Col-0 (**Figures 4H, I**). It can be speculated that the increased xylem area and the thickness of IF cell wall may be the main reasons for the increase in lignin content. These results strongly suggest that AtSYP71 regulates cell wall biosynthesis and dynamics.

### Stress response of *atsyp71-2* was significantly altered

**Figure 5:**
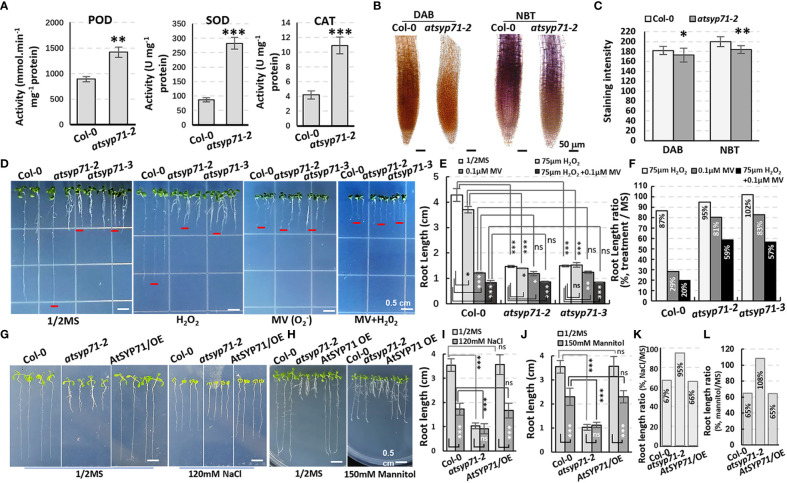
Alteration of ROS homeostasis and stress response in *atsyp71* mutants. **(A)** Statistics of antioxidase activities in roots of nine-day-old Col-0 and *atsyp71-2* seedlings. **(B)** DAB and NBT staining of roots of Col-0 and *atsyp71-2* nine-day-old seedlings. **(C)** Statistics of DAB and NBT staining intensities measured by Photoshop 2019. **(D)** Treatments on Col-0 and *atsyp71* nine-day-old seedlings with 75 μm H_2_O_2_, 0.1 μM MV, or 75 μm H_2_O_2 + _0.1 μM MV. n≈50. Three biological replicates per sample. **(E)** Statistics of root length shown representatively in **(D)**. **(F)** Statistics of ratio of root length in **(E)**. **(G)** Seven-day-old seedlings under 120 mM NaCl treatment. **(H)** Nine-day-old seedlings under 150 mM mannitol treatment. **(I, J)** Statistics of root length in **(G, H)**, respectively. n≈50. Three biological replicates per sample. **(K, L)** Statistics of ratio of root length in **(I, J)**, respectively. ns, no significance; *P < 0.05; **P < 0.01; ***P < 0.001. Student’s t test.

Transcriptome analysis indicates that in BP, a large amount of the DEGs were enriched in processes of stimuli/stress response (**Figure 2A**, red arrows), indicating AtSYP71 is essential for plant stress adaption. Therefore, we investigated the redox status of *atsyp71* mutants. The activities of antioxidases e.g. peroxidase (POD), superoxide dismutase (SOD) and superoxide catalase (CAT) significantly increased in *atsyp71-2* (**Figure 5A**). The DAB and NBT staining results revealed that H_2_O_2_ and ${\text{O}}_{2}^{-}$ levels in *atsyp71-2* decreased significantly compared with those in Col-0 (**Figures 5B, C**). These results indicate that ROS homeostasis was disturbed due to knockdown of *AtSYP71*. Then, we checked stress response of *atsyp71-2*. Under 75 μm H_2_O_2_ treatment, root length of Col-0 declined by about 13%, whereas that of *atsyp71-2* and *atsyp71-3* only declined by 2-5%. Under treatment of 0.1 μM methylviologen (MV, donor of ${\text{O}}_{2}^{-}$ ), root length of Col-0 declined dramatically by about 71%, however, that of *atsyp71* mutants only declined by 17-19%. While, under 0.1 μM MV + 75 μm H_2_O_2_ treatment, root length of both Col-0 and *atsyp71* mutants decreased more sharply, that of Col-0 declined by about 80%, and that of *atsyp71* mutants declined by 41-43% (**Figures 5D-F**). These results indicate that the ROS homeostasis was seriously interrupted in *atsyp71* mutants. Under 120 mM NaCl and 150 mM mannitol treatments, root length of Col-0 and *AtSYP71* OE seedlings reduced significantly, but that of *atsyp71-2* didn’t change obviously (**Figures 5G-J**). These results suggest that AtSYP71 modulates ROS homeostasis and affects plant stress response.

### Secretion of H^+^ in *atsyp71* mutant roots increased

**Figure 6:**
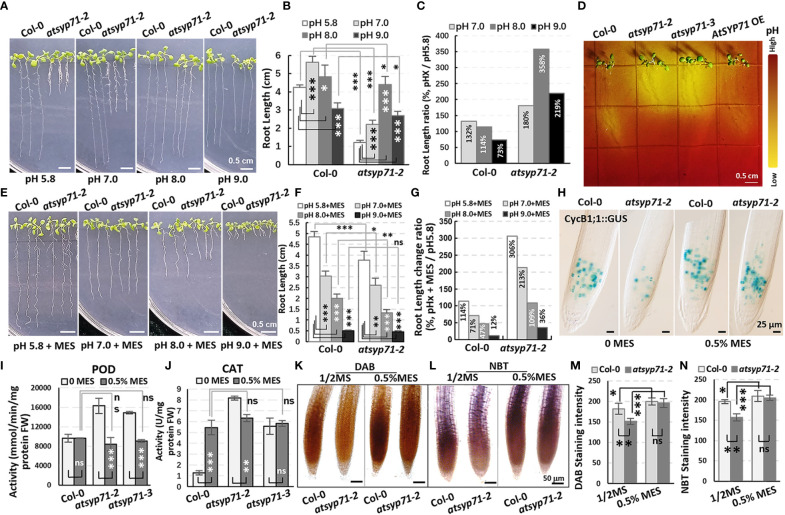
Alkali stress response altered in *atsyp71* mutants. **(A)** Phenotype of nine-day-old seedlings grown on 1/2MS medium with different pH value. **(B)** Statistics of root length of seedlings in **(A)**. **(C)** Statistics of ratio of root length in **(B)**. **(D)** Visualization of root acidification of Col-0, *atsyp71* mutants nd *AtSYP71* OE seedlings using the pH indicator, bromocresol purple. Five-day-old seedlings grown on 1/2MS medium (pH5.8) were transferred to 1/2MS medium (pH 6.8) containing 0.006% (w/v) bromocresol purple, and photographed three-day after transfer. **(E)** Phenotype of nine-day-old seedlings grown on 1/2MS medium containing 0.5% MES with different pH value. **(F)** Statistics of root length of seedlings in **(E)**. **(G)** Statistics of ratio of root length in **(F)**. **(H)** Nomarski images of *CycB1;1::GUS*-expressing cells in roots of nine-day-old Col-0 and *atsyp71-2* seedlings. **(I, J)** POD **(H)** and CAT **(I)** activities of nine-day-old seedlings grown on 1/2MS medium (pH5.8) with or without 0.5% MES. **(K, L)** DAB **(K)** and NBT **(L)** staining of nine-day-old seedlings grown on 1/2MS medium (pH5.8) with or without 0.5% MES. **(M, N)** Statistics of staining intensities in **(K)** and **(L)**. ns, no significance; *P < 0.05; **P < 0.01; ***P < 0.001. Student’s t test.

Since GO Enrichment analysis revealed a DEG enrichment in Response to acid chemical process (**Figure 2A**, green arrow), we further investigated growth of *atsyp71* mutants under different pH value. In 1/2MS medium with pH7 condition, the root length of both Col-0 and *atsyp71-2* increased significantly. Under pH8 condition, root growth of Col-0 largely recovered, however, root growth of *atsyp71-2* increased more significantly than that at pH5.8, and at pH9, root growth of Col-0 was inhibited significantly, however, root length of *atsyp71-2* was still significantly higher than that at pH5.8 (**Figures 6A-C**), means that *atsyp71-2* root growth was better under alkaline conditions. Therefore, we speculated that *atsyp71-2* roots probably secreted more acidic substances to the apoplast to alleviate the inhibition of root growth under alkali stress. To verify our speculation, bromocresol purple, a pH indicator, was used to detect the acidification of the medium growing *atsyp71-2* mutants and Col-0. As expected, the medium around *atsyp71-2* and *atsyp71-3* roots showed higher acidification than that around Col-0 and *AtSYP71* OE roots (**Figure 6D**). These results demonstrate that AtSYP71 may affect root pH homeostasis. Then, we added MES [2-(N-morpholino) ethanesulfonic acid], a broadly used Good’s buffer, to 1/2MS medium to observe its effects on root growth. To obtain the appropriate MES concentration for treatment, 0.1% and 0.5% MES were tested. It was found that at 0.5% MES condition, root length of *atsyp71* mutants was recovered better that that at 0.1% MES condition (**Supplementary Figure 4**). Thus, 0.5% MES was used for subsequent experiments.

Under pH5.8 with 0.5% MES condition, the root length of both Col-0 and *atsyp71-2* increased significantly compared with that at pH5.8, while, the root elongation of *atsyp71-2* (by 306%) was more significant than that of Col-0 (by 114%). At pH7 and pH8 with 0.5% MES, the root growth of Col-0 was inhibited and the root length was significantly shorter than that at pH5.8 (by 71% and 47%, respectively), whereas root length of *atsyp71-2* was still significantly longer than that at pH5.8 (by 216% and 109%, respectively). And under pH9 with 0.5% MES, root growth of both Col-0 and *atsyp71-2* was inhibited seriously (**Figures 6E-G**). To study root apical meristem (RAM) activity under adding MES condition, we observed the mitotic marker CycB1;1::GUS which was introduced into *atsyp71-2* by crossing. The GUS staining results revealed that under pH5.8, the RAM activity in *atsyp71-2* roots was seriously inhibited compared with that in Col-0, but when added 0.5% MES, the RAM activity in *atsyp71-2* was restored and was close to the level in Col-0 (**Figure 6H**). These results suggest that AtSYP71 regulates root development *via* modulating pH homeostasis. To figure out whether pH homeostasis is connected with ROS homeostasis, we checked ROS level in *atsyp71* roots under MES condition. First, we determinated antioxidase activities of the seedlings grown on 1/2MS medium (pH5.8) with or without 0.5% MES. At pH5.8 with 0.5% MES, the POD activities of Col-0 did not change significantly, whereas that of *atsyp71* mutants decreased significantly compared with that at pH5.8. There was no significant difference in POD activities between Col-0 and *atsyp71* mutants after adding 0.5% MES (**Figure 6I**). On the other hand, in the presence of 0.5% MES, the CAT activities of Col-0 increased significantly, while, that of *atsyp71-2* reduced significantly compared with that without MES. And there was no significant difference in CAT activities between Col-0 and *atsyp71* mutants after adding 0.5% MES (**Figure 6J**). The DAB and NBT staining revealed that under 0.5% MES condition, H_2_O_2_ and ${\text{O}}_{2}^{-}$ levels in both Col-0 and *atsyp71-2* increased significantly, but there was little difference between Col-0 and *atsyp71-2* (**Figures 6K-N**). These results indicate that MES buffered the excess H^+^ produced by *atsyp71-2* roots and consequently restored the ROS level, suggesting that AtSYP71 affects ROS homeostasis and root development *via* regulating pH homeostasis.

### Secretion was disturbed in *atsyp71-2* mutant

**Figure 7:**
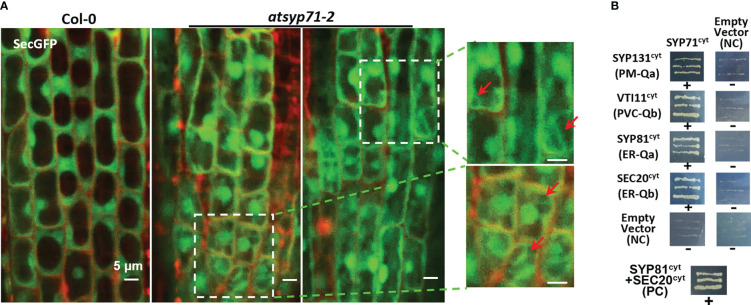
AtSYP71 regulated secretion. **(A)** Confocal images of SecGFP in Col-0 and *atsyp71-2* with PI staining. The magnified images on the right are the part with white boxes. Arrows indicate the cytoplasmic localization of Sec-GFP. **(B)** Yeast two hybrid analysis of AtSYP71 interactors. Yeast strain AH109 was transformed with the paired constructs as shown. Transformants were streaked onto SD/_Leu/_Trp/_His/_Ade medium. AtSYP81/pGADT7 vs AtSec20/pGBKT7 served as a positive control. Each construct and its corresponding empty vector were used as negative controls. cyt, cytoplasmic fragment; FL, full length. +, has an interaction; -, no interaction.

Since cell wall biosynthesis and acid substance secretion are regulated by secretion pathway, we observed the localization of the secretory marker SecGFP (Batoko et al., 2000; Leucci et al., 2007), in *atsyp71-2* root cells. Confocal images revealed that in *atsyp71-2* root cells, SecGFP displayed apparent cytoplasmic localization which was absent in Col-0 (**Figure 7A**, arrows), indicating that part of SecGFP was blocked in secretion pathway, suggesting that AtSYP71 plays crucial role on secretion.

Then, we identified AtSYP71- associating proteins. AtSYP71 is reported to localize on the ER, endosome, plasma membrane and cell plate (Alexandersson et al., 2004; Marmagne et al., 2004; Mongrand et al., 2004; Uemura et al., 2004; Morel et al., 2006), suggesting its multiple functions. During cytokinesis, AtSYP71 associates with Qa-SNARE KNOLLE, Qb-SNARE NPSN11, and R-SNARE VAMP721/722 to form a tetrameric SNARE complex in cell plate (El Kasmi et al., 2013). To further identify AtSYP71 partners, we generated *myc-AtSYP71/atsyp71-2* lines by crossing *myc-AtSYP71* (*AtSYP71OE*) line with *atsyp71-2* mutant. Immunoblot analysis indicates recovery of AtSYP71 protein level and root length compared with *atsyp71-2* (**Supplementary Figure 5**), implying that myc-AtSYP71 proteins were functional. Then, we performed pull down of myc-AtSYP71 and identified PM-localized Qa-SNARE SYP131, SYP121 and SYP122, PM-Qb-SNARE NPSN11 and NPSN12, prevacuolar compartment (PVC)-localized Qb-SNARE VTI11 and SYP22 (**Table 1**). Among these factors, SYP121/SYP122, NPSN11/NPSN12, and SYP22 are demonstrated to be co-immunoprecipitated with AtSYP71 (Fujiwara et al., 2014). For the rest unclarified factors, VTI11 and SYP131, we performed yeast two hybrid (Y2H) analysis. As expected, Y2H results confirmed the interactions of AtSYP71 with VTI11 and SYP131, respectively (**Figure 7B**). AtSYP71 is also localized at the ER, but the pull down products did not include ER-localized SNAREs, suggesting the interaction of AtSYP71 with the ER-SNARE was minor. Therefore, we performed pull down-LC-MS/MS analysis using myc-tagged ER-Qa-SNARE *AtSYP81*-overexpressing plants. The elution products include AtSYP71, and ER-Qb-SNARE SEC20, as well as the PM- and PVC-localized AtSYP71 partner proteins (**Table 2**). Y2H confirmed the interactions of AtSYP71 with AtSYP81 and SEC20, respectively (**Figure 7B**). These results suggest that during interphase, AtSYP71 may form distinct SNARE complexes with different set of SNAREs to mediate membrane fusion of multiple steps in secretory or vacuole-targeting pathways, respectively.

**Table 1:** Pull down-LC-MS/MS analysis identified AtSYP71-associated proteins related to vesicle transport using myc-AtSYP71-overexpressing plants.

Gene ID	Name	Description		hit_1		matches_1 (myc- AtSYP71/WT)		score_1		hit_2		matches_2 (myc- AtSYP71/WT)		score_2		hit_3		matches_3 (myc- AtSYP71/WT)		score_3	
AT3G09740	SYP71	ER, Qc-SNARE		25		51/0		35.21		28		44/0		22.5		1		115/0		28.84	
AT3G03800	SYP131	PM, Qa-SNARE		19		5/0		25.03		—		—		—		—		—		—	
AT3G11820	SYP121	PM, Qa-SNARE		303		16/0		32.06		19		9/0		32.81		—		—		—	
AT3G52400	SYP122	PM, Qa-SNARE		1917		2/0		38.84		304		2/0		21.84		19		5/0		25.85	
AT2G35190	NPSN11	PM, Qb-SNARE		538		8/0		19.18		514		7/0		33.28		29		17/0		32.32	
AT1G48240	NPSN12	PM, Qb-SNARE		538		8/0		16.72		514		5/0		19.33		29		10/0		17.76	
AT5G39510	VTI11	TGN/PVC/vacuole, Qb-SNARE		1147		4/0		18.4		968		5/0		54.03		—		—		—	
AT5G46860	SYP22	PVC/vacuole, Qb-SNARE		1269		6/0		26.34		2708		4/0		19.38		—		—		—	

Hit, peptide_hit_number; score, peptide score. Identification of SYP71 itself only in transgenic plants indicates the reliability of the experiments.

**Table 2:** Pull down-LC-MS/MS analysis identified AtSYP81-associated proteins which are related to vesicle trafficking using myc-AtSYP81-overexpressing plants.

Gene ID	Name	Description	hit_1	matches_1 (myc- AtSYP71/WT)	score_1	hit_2	matches_2 (myc- AtSYP71/WT)	score_2
AT1G51740	SYP81	ER, Qa-SNARE	10	86/0	19.37	2	10/0	16.44
AT3G24315	SEC20	ER, Qb-SNARE	62	29/0	33.5	—	—	—
AT3G09740	SYP71	ER, Qc-SNARE	1052	11/0	28.14	30	33/0	21.36
AT3G11820	SYP121	PM, Qa-SNARE	3261	4/0	32.65	66	14/0	17.66
AT3G52400	SYP122	PM, Qa-SNARE	228	4/0	15.44	—	—	—
AT2G35190	NPSN11	PM, Qb-SNARE	2310	1/0	45.6	87	12/0	15.13
AT1G48240	NPSN12	PM, Qb-SNARE	87	8/0	23.28	—	—	—
AT5G39510	VTI11	TGN/PVC/vacuole, Qb-SNARE	935	5/0	46.48	145	6/0	32.43
AT5G46860	SYP22	PVC/vacuole, Qb-SNARE	912	5/0	25.94	—	—	—

Hit, peptide_hit_number; score, peptide score. Identification of SYP81 itself only in transgenic plants indicates the reliability of the experiments.

## Discussion

### AtSYP71 affects plant development *via* regulating secretory pathway

AtSYP71 has an unusual multiple localization on the ER, endosome, PM and cell plate, the compartments in the secretory pathways (Suwastika et al., 2008; El Kasmi et al., 2013). It is clarified that AtSYP71 binds with Qa-KNOLLE, Qb-SNARE NPSN11 and R-SNAREs VAMP721 and VAMP722 to form a SNARE complex regulating cytokinesis (El Kasmi et al., 2013). AtSYP71 is also demonstrated to be co-immunoprecipitated with SYP121/SYP122, NPSN11/NPSN12, and SYP22 (Fujiwara et al., 2014). In this study, we found that in addition to the mentioned factors, AtSYP71 interacted with PVC-localized SNARE, VTI11, suggesting that AtSYP71 may be also involved in vacuole-targeting pathway. Considering importance of AtSYP71 functions, deficiency of AtSYP71 will definitely seriously affect AtSYP71-dependent vesicle trafficking and organelle functions. Lethality of the knock-out mutant *atsyp71-1* (**Figure 1C**) resembled the phenotype of *syp71^amiR^* and *npsn11 syp71^amiR^*, the cytokinesis defective mutants (El Kasmi et al., 2013). The failure of morphogenesis may be due to the blocked delivery of materials required for cell plate formation, implying essential role of AtSYP71 in secretion and development in *Arabidopsis*. On the contrary, *Ljsyp71* mutants grow similarly to wild-type plant when supplied with combined nitrogen. *OsSYP71* and *TaSYP71* deficient mutants didn’t show obvious phenotypes under normal growth conditions (Bao et al., 2012; El Kasmi et al., 2013). These suggest that *LjSYP71*, *OsSYP71* and *TaSYP71* are not essential for plant growth and development. This probably because that SYP71 orthologues in different species gain functional division during evolutionary.

The knock-down mutants, *atsyp71-2* and *atsyp71-3*, also displayed developmental defects. While, the short root phenotype was rescued by alkali pH and 0.5% MES which buffers H^+^ (**Figure 6**). The acidification of *atsyp71-2* and *atsyp71-3* rhizosphere indicate that the pH homeostasis was affected, suggesting that homeostasis of PM-localized ion channels and other PM-residents may also be disturbed. For example, SLAH3 mainly inhibit the inward-rectifying K^+^ channel KAT1 by protein-protein interaction and consequently prevent stomatal opening (Zhang et al., 2016). CIPK3 regulates K^+^ homeostasis through activating vacuolar K^+^ efflux to the cytoplasm (Tang et al., 2020). Transcriptional levels of these two genes altered significantly in *atsyp71-2* (**Supplementary Figure 6A**), suggesting AtSYP71 effects on ion homeostasis. The fundamental reason of unbalanced homeostasis in the mutants was the block of secretory and cycling pathways which maintain homeostasis of PM-localized ion channels and other residents, as well as exocytosis of cell wall biosynthetic materials which led to changes of cell wall structure and components (**Figure 4**; **Supplementary Figure 3**).

Knockdown of *AtSYP71* definitely impacted the organelle functions. The ER and the Golgi apparatus are responsible for protein glycosylation. Thus, post transcriptional modification of the glycoproteins, e.g. laccases, PRXs and AGPs, were likely disrupted in *atsyp71* mutants. Transcriptome analysis indicate that the expression of *PRX4/15/25/37/49/52/53/54/62*, *LAC12* and *AGP1* genes decreased significantly (**Figure 3**), and the protein level of AGPs were also reduced significantly in *atsyp71-2* (**Figure 4D**). Furthermore, the expression of UDP-glucosyltransferase, UGT74E2 and AT2G18560, the cell wall biosynthesis regulators, were also altered significantly (**Supplementary Figure 6B**). These changes validate effects of AtSYP71 on the regulatory machinery of cell wall biosynthesis. In addition, expression of many cell wall dynamics regulatory genes were also affected in *atsyp71-2*. For example, EXPA proteins are responsible for cell wall extension and induce stress relaxation and extension of cell wall in a pH-dependent manner (Cosgrove, 2000). XTH proteins catalyze molecular grafting and/or hydrolysis of cell wall xyloglucans (Jiang et al., 2020), and their function are pH-dependent (Cosgrove, 2000). LTP2 plays a role in maintaining the integrity of the cuticle-cell wall interface (Julke and Ludwig-Muller, 2015). PP2-B13, is a phloem protein 2 (PP2)-like protein. PP2 is a component of the phloem protein bodies, directly bind with the chitin cell wall and play important roles in defense and wound healing (Beneteau et al., 2010). BGLU25 belongs to beta-glucosidase family which is involved in cellulose degradation (Roepke and Bozzo, 2015; Han et al., 2020). Expression of these genes altered significantly (**Figure 3**), suggesting AtSYP71 regulatory role on cell wall dynamics. Among the genes, EXPA and XTH proteins function in a pH-dependent manner (Cosgrove, 2000; Jiang et al., 2020), and excessive acidification of *atsyp71* mutants likely destroyed the optimal working pH of these kinds of proteins and resulted in defects on cell elongation.

### AtSYP71 regulates ROS homeostasis partially *via* controlling pH homeostasis

Abiotic stresses induce high level of ROS production. The ROS scavenging system includes enzymatic and non-enzymatic antioxidant (You and Chan, 2015). The enzymatic antioxidants include SOD, CAT, POD, APX, glutathione peroxidase (GPX), glutathione-S-transferase (GST) etc. which reduce ${\text{O}}_{2}^{\cdot -}$ and H_2_O_2_ (Gill and Tuteja, 2010; Nadarajah, 2020). SODs have diverse forms containing distinct metal ions such as Fe, Mn or Cu/Zn, and are distributed in the apoplast (Kim et al., 2008), cytosol, chloroplasts, mitochondria and peroxisomes (Pilon et al., 2011). Respiratory burst oxidase homologues (RBOHs), the plant NADPH oxidases (NOXs), are localized on the PM and convert oxygen to ${\text{O}}_{2}^{\cdot -}$ in the apoplast (Segal, 2016). Under stress conditions, ${\text{O}}_{2}^{-}$ /H_2_O_2_ production occurs in cytoplasmic vesicles derived from the PM or ER (Leshem et al., 2006), and RBOHD is internalized into PM-derived vesicles (Hao et al., 2014). Oxidation also occurs inside the ER lumen to promote protein folding depending on disulfide bonds formation by a FAD-containing ER oxidase (ERO) and protein disulfide isomerases (PDIs) (Bulleid, 2012). The homeostasis of the organelles determines ROS homeostasis, thus, dysfunction of the ER and PM in *atsyp71-2* definitely affected ROS homeostasis (**Figure 5**).

Apoplastic H_2_O_2_ influences PRX-mediated lignification and cross-linking of cell wall polymers (Marjamaa et al., 2009; Shigeto et al., 2015). In *atsyp71-2*, in addition to *PRXs*, expression of many redox-related genes altered significantly, such as *AOC3* (endothelial amine oxidase), *POX1* (proline oxidase family FAD-linked oxidoreductase), *AT4G20830* and *AT5G44400* (FAD linked oxidase homologues), *LAC1* (multicopper oxidase) and *AT1G31710* (Copper amine oxidase homologue) (**Supplementary Figure 6B**). The changes in these enzyme activities may be one of the reason of unbalanced ROS homeostasis. And the significantly increased lignin content was probably the consequence of the unbalanced ROS homeostasis in *atsyp71-2*.

The non-enzymatic antioxidants contain ascorbic acids, α-tocopherol, flavonoids, phenolic compounds, glutathione, carotenoids and lipids, which mitigate oxidative damage by their antioxidant activities through utilization of H_2_O_2_ (Duan et al., 2012; Kim et al., 2020b). Flavonoids distribute widely in plants and are one of the most bioactive plant secondary metabolites. Flavonoids are synthesized on the ER surface and uploaded into the ER lumen, and subsequently transported to the vacuole or is secreted to the apoplast (Zhao and Dixon, 2010; Falcone Ferreyra et al., 2012; Zhao, 2015). The PM-localized ABCG transporters mediated flavonoid efflux (Banasiak et al., 2013; Liu et al., 2022b). In *atsyp71-2*, expression of ABCG1 decreased significantly (**Figure 3**); in response, content of flavonoids decreased significantly (**Figure 4B**). Dysfunction of the ER and PM in *atsyp71-2* definitely affected flavonoids production and secretion, which subsequently affected stress response of the mutants.

## Data availability statement

The original contributions presented in the study are publicly available. This data can be found here: The National Center for Biotechnology Information with Bioproject ID PRJNA971388 https://www.ncbi.nlm.nih.gov/bioproject/PRJNA971388.

## Author contributions

LL conceived and supervised the project. LL, HZ, JZ and XK designed the experiments. HZ, JZ, XK, YL, XZ, MW, WL, YH, XTW, GTQ and ZZ generated materials and performed the experiments. GCQ performed the LC-MS/MS analysis. SL and XQW determinated the cell wall polysaccharides. LL, HZ, JZ and XK drafted the manuscript. LJ, XF and J-KZ cooperated and supervised the project.

## Supplementary material

Supplementary Figure 1 Overview of transcriptome analysis.

Supplementary Figure 2 Statistics of RT-qPCR analysis of DEG expression levels in *atsyp71-3.*

Supplementary Figure 3 Detection of contents of AGPs and XyG.

Supplementary Figure 4 Effects of different MES concentration on seedling development.

Supplementary Figure 5 myc-AtSYP71 protein has functionality.

Supplementary Figure 6 Transcriptomic analysis of ion channels **(A)** and redox-related genes **(B)**.

Supplementary Table 1 Original transcriptomic data of *atsyp71-2* vs Col-0.

Supplementary Table 2 Information of DEGs enriched in cell wall biosynthesis- and dynamics-related pathways.

Supplementary Table 3 Primer information used in this study.
