# Supplementary material for: Syntaxin of plants71 plays essential roles in plant development and stress response via regulating pH homeostasis
Source: Front Plant Sci. 2023 Jun 5;14:1198353. doi: 10.3389/fpls.2023.1198353 (PMC10277689; doi:10.3389/fpls.2023.1198353)
Supplement: Supplementary Figure 1 — Overview of transcriptome analysis. [file DataSheet_1.pdf]

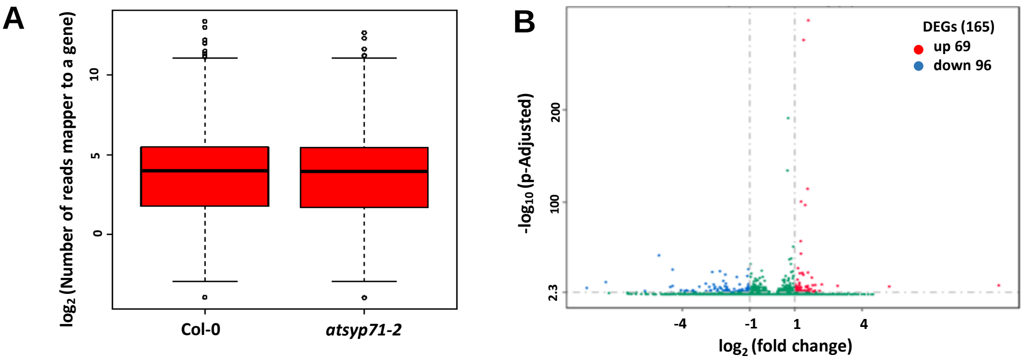

Supplementary Figure 1. Overview of transcriptome analysis. (A) Transcriptome data quality analysis. **(B)** Volcano plots of DEGs. X axis represents $\log_2$ fold change. Y axis represents $-\log_{10}$ significance. Red points, up-regulated DEGs; blue points, down-regulated DEGs; green dots, genes with no significance.

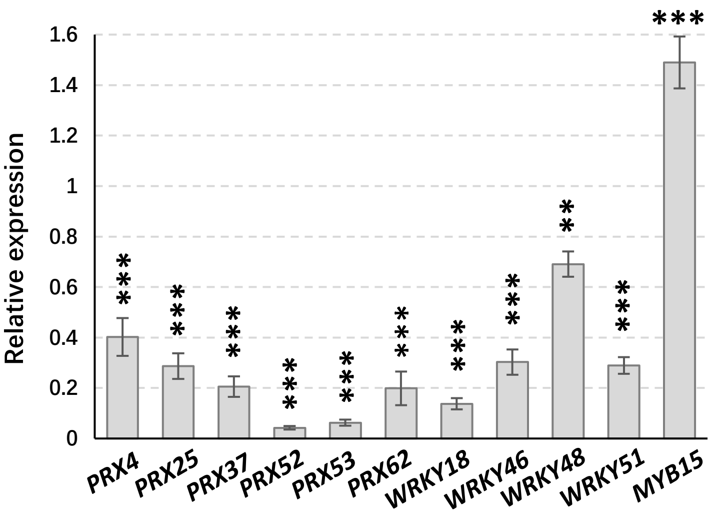

Supplementary Figure 2. Statistics of RT-qPCR analysis of DEG expression levels in *atsyp71-3*. The total RNA was extracted from roots of nine-day-old seedlings. Three independent experiments performed with two technical replicates per sample. **, $P < 0.01$; ***, $P < 0.001$; Student's t-test.

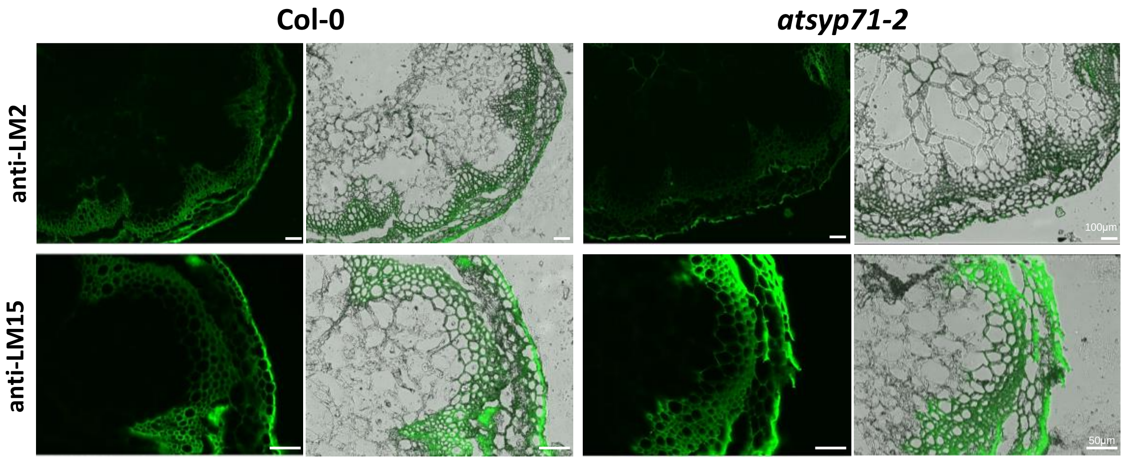

Supplementary Figure 3. Detection of contents of AGPs and XyG. Immunolabeling of AGPs with anti-LM2 antibody (upper panel) and XXXG XyG with anti-LM15 antibody (lower panel), respectively, using slice of cross section of the first stem segments from ten-week-old Col-0 and *atsyp71-2* plants.

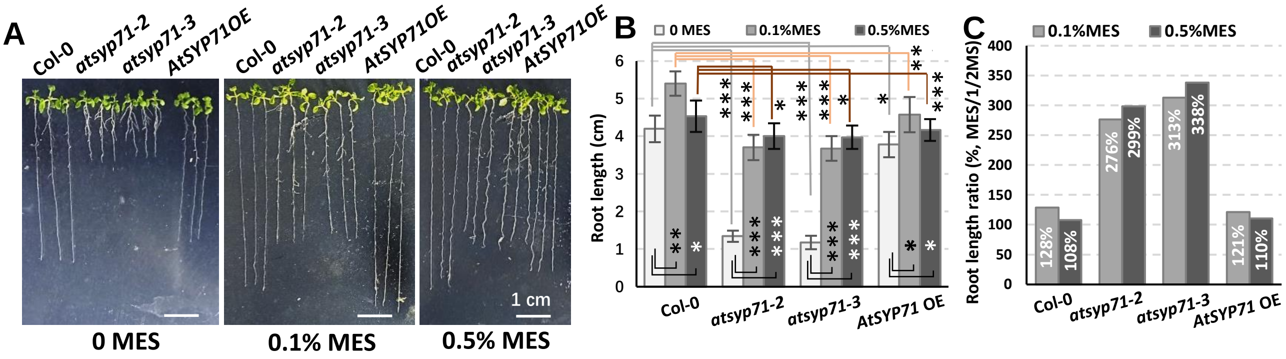

Supplementary Figure 4. Effects of different MES concentration on seedling development. (A) Phenotype of nine-day-old seedlings grown on 1/2MS medium containing 0.1% MES and 0.5% MES. **(B)** Statistics of root length of seedlings in **(A)**. **(C)** Statistics of ratio of root length in **(A)**.

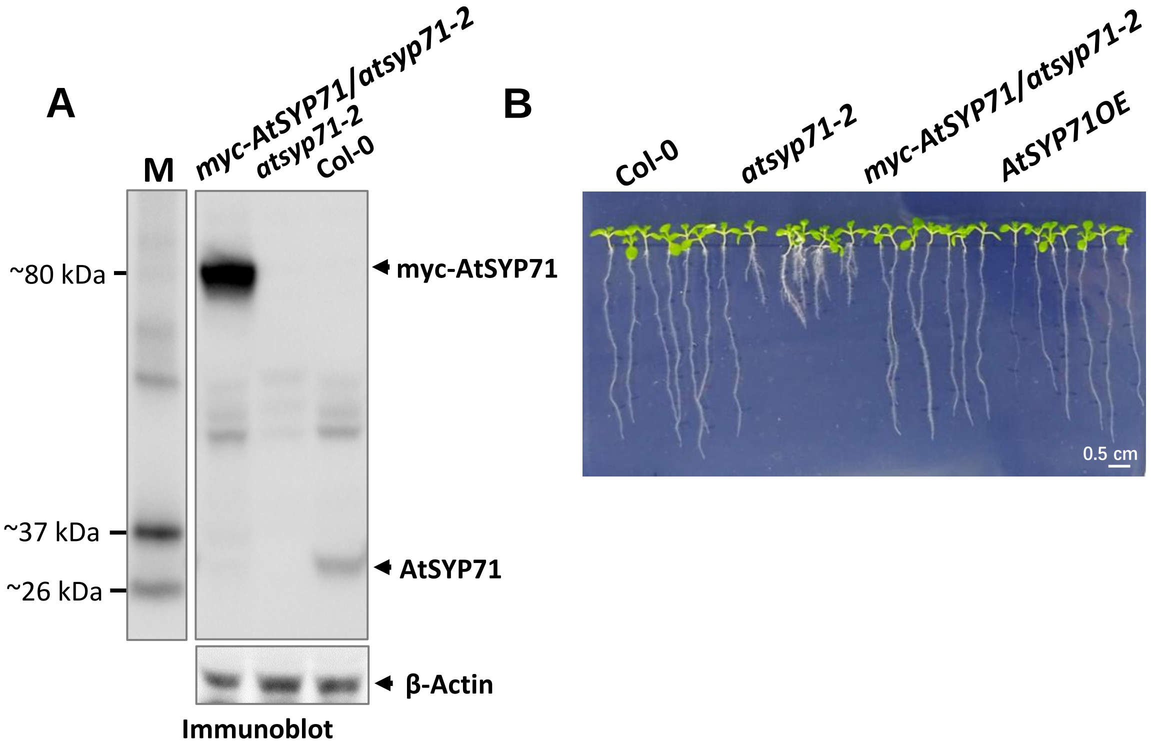

Supplementary Figure 5. myc-AtSYP71 protein has functionality. (A) Immunoblot analysis of AtSYP71 protein abundance with anti-AtSYP71 antibodies using seven-day-old seedlings of Col-0, *atsyp71-2*, *AtSYP71OE*, and *myc-AtSYP71/atsyp71-2*. β-Actin was used as endogenous control. Three independent experiments were performed, showing similar results. M, marker. **(B)** Phenotypes of seven-day-old seedlings of the indicated lines.

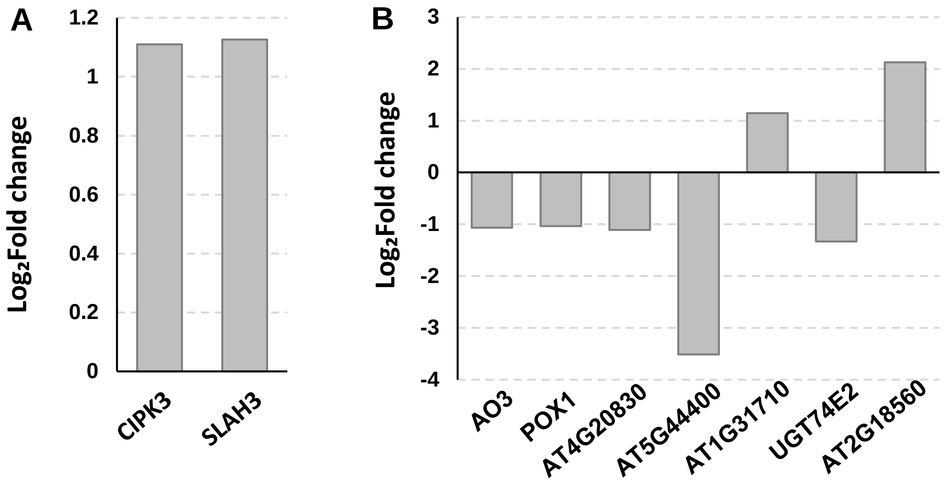

Supplementary Figure 6. Transcriptomic analysis of ion channels **(A)** and redox-related genes **(B)**.
