# Supplementary material for: Syntaxin of plants71 plays essential roles in plant development and stress response via regulating pH homeostasis
Source: Front Plant Sci. 2023 Jun 5;14:1198353. doi: 10.3389/fpls.2023.1198353 (PMC10277689; doi:10.3389/fpls.2023.1198353)
Supplement: Supplementary Table 2 — Information of DEGs enriched in cell wall biosynthesis- and dynamics-related pathways. [file Table_2.docx]

**Supplementary Table 2. DEGs enriched in cell wall biosynthesis- and dynamics-related pathways.**

| **Gene name** | **Gene ID** | **Function** | **References** |
| --- | --- | --- | --- |
| *PRX4* | AT1G14540 | **Involved** in cell wall lignification. | Fernández-Pérez et al., 2015 |
| *PRX15* | AT2G18150 | — | — |
| *PRX25* | AT2G41480 | **PRX2**, PRX25 and PRX71, are involved in stem lignification. | Shigeto et al., 2015 |
| *PRX37* | AT4G08770 | **Involved** in cell wall biosynthesis. | Pedreira et al., 2011 |
| *PRX49* | AT4G36430 | — | — |
| *PRX52* | AT5G05340 | **Involved** in the synthesis of S units in interfascicular fibers in lignification. | Fernández-Pérez et al., 2015 |
| *PRX53* | AT5G06720 | **Likely** involved in cross-linking of cell wall compounds. | Jin et al., 2011 |
| *PRX54* | AT5G06730 | — | — |
| *PRX62* | AT5G39580 | **PRX62** and PRX69 are key apoplastic PRXs that modulate ROS-homeostasis and cell wall extension-insolubilization linked to Root Hair elongation at low temperature. | Pacheco et al., 2022 |
| *BGLU25* | AT3G03640 | **Involved** in the carbohydrate metabolism and glycosyl compound metabolic process. | Xu et al., 2004 |
| *BGAL8* | AT2G28470 | **Located** in the cell walls and plays a role on cell wall remodeling and expansion. | Chandrasekar and van der Hoorn, 2016 |
| *SCPL12* | AT2G22920 | — | — |
| *LTPG1* | AT1G27950 | **LTPG1** and LTPG2 contribute to the accumulation of cuticular lipids. | Fahlberg et al., 2019 |
| *LTP2* | AT2G38530 | **Plays** a role in maintaining the integrity of the cuticle-cell wall interface. | Bard et al., 2016 |
| *LTP5* | AT3G51600 | **A** small secreted peptide from both pollen and the pistil, plays a role in pollen tube tip growth and in pistil function. | Chae et al., 2009 |
| *EXPA1* | AT1G69530 | **A** cell wall modifying enzyme which take part in cell growth and cell wall disassembly during syncytium formation. | Wieczorek et al., 2006 |
| *EXPA4* | AT2G39700 | **A** putative expansin. Involved in the formation of nematode-induced syncytia in roots of *Arabidopsis thaliana*. | Liu et al., 2021 |
| *XTH20* | AT5G48070 | **A** putative xyloglucan endotransglycosylase/hydrolase expressed primarily in the main and lateral roots, is involved in cell proliferation in incised inflorescence stems. | Pitaksaringkarn et al., 2014 |
| *XTH22* | AT5G57560 | **Regulates** plant growth by disrupting the cell wall homeostasis in *Arabidopsis* under boron deficiency. | Zhang et al., 2022 |
| *GSTF6* | AT1G02930 | **Catalyzes** GSH (IAN) formation, GSH is determined to be intermediates within the camalexin biosynthetic pathway, thereby participating in camalexin biosynthesis. | Su et al., 2011 |
| *CYP706A1* | AT4G22690 | — | — |
| *ABCG1* | AT2G39350 | **Transport** of longer chain aliphatic monomers from the cytoplasm to the apoplastic space during root suberin formation. | Shanmugarajah et al., 2019 |
| *WRKY18* | AT4G31800 | **A** regulator for modulating ABA signaling and influencing ABA-regulated plant growth and abiotic stress responses. | Chen et al., 2010 |
| *WRKY46* | AT2G46400 | **Plays** roles in regulating plant responses to drought. | Chen et al., 2017 |
| *WRKY48* | AT5G49520 | **Plays** a role in plant responses to abiotic stresses. | Xing et al., 2008 |
| *WRKY51* | AT5G64810 | **Mediates** both SA- and low-18:1-dependent repression of JA signaling. | Gao et al., 2011 |
| *MYB15* | AT3G23250 | **Required** for the activation of lignin biosynthesis genes and consequently lignin formation during effector-triggered immune responses. | Kim et al., 2020 |
| *HRS1* | AT1G13300 | **Prevent** the excessive accumulation of N by transducing the nitrogen saturation signal. | Li et al., 2021 |
| *JAL22* | AT2G39310 | **An** inhibitor-type lectin, regulate the size of the PYK10, a major protein of ER bodies complex antagonistically. | Nagano et al., 2008 |
| *BBE20/*  *OGOX1* | AT4G20830 | **An** oligogalacturonide (OG) oxidase, is involved in plant immunity. OGs, a major component of pectin, are a well-known class of damage-associated molecular patterns (DAMPs) that activate immunity and protect plants against microbes. | Benedetti et al., 2018 |
| *BBE26* | AT5G44400 | — | — |
| *BBE11* | AT1G30730 | — | — |
| *PP2-B13* | AT1G56240 | **A** phloem protein 2-like protein, belongs to F-box-like domain superfamily. PP2 is component of the phloem protein bodies found in sieve elements. PP2 proteins can directly bind with the chitin cell wall and play important roles in defense against pathogens, photoassimilate transport and wound healing. | Bobbili et al., 2018; Guo et al., 2018; Akash et al., 2021 |
| *AGP1* | AT5G64310 | **Involved** in pollen grain competence to initiate pollen tube growth. | Pereira et al., 2014 |
| *LAC12* | AT5G05390 | **Plays** a role in root-to-shoot Fe partitioning and in maintaining growth on Fe-deficient substrates. | Bernal and Krämer, 2021 |

**Akash, Parida AP, Srivastava A, Mathur S, Sharma AK, Kumar R** 2021 Identification, evolutionary profiling, and expression analysis of F-box superfamily genes under phosphate deficiency in tomato. Plant Physiol Biochem **162:** 349-362

**Bard GC, Zottich U, Souza TA, Ribeiro SF, Dias GB, Pireda S, Da Cunha M, Rodrigues R, Pereira LS, Machado OL, Carvalho AO, Gomes VM** 2016 Purification, biochemical characterization, and antimicrobial activity of a new lipid transfer protein from Coffea canephora seeds. Genet Mol Res **15**

**Benedetti M, Verrascina I, Pontiggia D, Locci F, Mattei B, De Lorenzo G, Cervone F** 2018 Four Arabidopsis berberine bridge enzyme-like proteins are specific oxidases that inactivate the elicitor-active oligogalacturonides. Plant J **94:** 260-273

**Bernal M, Krämer U** 2021 Involvement of Arabidopsis Multi-Copper Oxidase-Encoding LACCASE12 in Root-to-Shoot Iron Partitioning: A Novel Example of Copper-Iron Crosstalk. Front Plant Sci **12:** 688318

**Bobbili KB, Pohlentz G, Narahari A, Sharma K, Surolia A, Mormann M, Swamy MJ** 2018 Coccinia indica agglutinin, a 17kDa PP2 like phloem lectin: Affinity purification, primary structure and formation of self-assembled filaments. Int J Biol Macromol **108:** 1227-1236

**Chae K, Kieslich CA, Morikis D, Kim SC, Lord EM** 2009 A gain-of-function mutation of Arabidopsis lipid transfer protein 5 disturbs pollen tube tip growth and fertilization. Plant Cell **21:** 3902-3914

**Chandrasekar B, van der Hoorn RA** 2016 Beta galactosidases in Arabidopsis and tomato - a mini review. Biochem Soc Trans **44:** 150-158

**Chen H, Lai Z, Shi J, Xiao Y, Chen Z, Xu X** 2010 Roles of arabidopsis WRKY18, WRKY40 and WRKY60 transcription factors in plant responses to abscisic acid and abiotic stress. BMC Plant Biol **10:** 281

**Chen J, Nolan TM, Ye H, Zhang M, Tong H, Xin P, Chu J, Chu C, Li Z, Yin Y** 2017 Arabidopsis WRKY46, WRKY54, and WRKY70 Transcription Factors Are Involved in Brassinosteroid-Regulated Plant Growth and Drought Responses. Plant Cell **29:** 1425-1439

**Fahlberg P, Buhot N, Johansson ON, Andersson MX** 2019 Involvement of lipid transfer proteins in resistance against a non-host powdery mildew in Arabidopsis thaliana. Mol Plant Pathol **20:** 69-77

**Fernández-Pérez F, Pomar F, Pedreño MA, Novo-Uzal E** 2015 The suppression of AtPrx52 affects fibers but not xylem lignification in Arabidopsis by altering the proportion of syringyl units. Physiol Plant **154:** 395-406

**Fernández-Pérez F, Vivar T, Pomar F, Pedreño MA, Novo-Uzal E** 2015 Peroxidase 4 is involved in syringyl lignin formation in Arabidopsis thaliana. J Plant Physiol **175:** 86-94

**Gao QM, Venugopal S, Navarre D, Kachroo A** 2011 Low oleic acid-derived repression of jasmonic acid-inducible defense responses requires the WRKY50 and WRKY51 proteins. Plant Physiol **155:** 464-476

**Guo P, Zheng Y, Peng D, Liu L, Dai L, Chen C, Wang B** 2018 Identification and expression characterization of the Phloem Protein 2 PP2 genes in ramie Boehmeria nivea L. Gaudich . Sci Rep **8:** 10734

**Jin J, Hewezi T, Baum TJ** 2011 Arabidopsis peroxidase AtPRX53 influences cell elongation and susceptibility to Heterodera schachtii. Plant Signal Behav **6:** 1778-1786

**Kim SH, Lam PY, Lee MH, Jeon HS, Tobimatsu Y, Park OK** 2020 The Arabidopsis R2R3 MYB Transcription Factor MYB15 Is a Key Regulator of Lignin Biosynthesis in Effector-Triggered Immunity. Front Plant Sci **11:** 583153

**Li Q, Zhou L, Li Y, Zhang D, Gao Y** 2021 Plant NIGT1/HRS1/HHO Transcription Factors: Key Regulators with Multiple Roles in Plant Growth, Development, and Stress Responses. Int J Mol Sci **22**

**Liu W, Xu L, Lin H, Cao J** 2021 Two Expansin Genes, AtEXPA4 and AtEXPB5, Are Redundantly Required for Pollen Tube Growth and AtEXPA4 Is Involved in Primary Root Elongation in Arabidopsis thaliana. Genes Basel **12**

**Nagano AJ, Fukao Y, Fujiwara M, Nishimura M, Hara-Nishimura I** 2008 Antagonistic jacalin-related lectins regulate the size of ER body-type beta-glucosidase complexes in Arabidopsis thaliana. Plant Cell Physiol **49:** 969-980

**Pacheco JM, Ranocha P, Kasulin L, Fusari CM, Servi L, Aptekmann AA, Gabarain VB, Peralta JM, Borassi C, Marzol E, Rodríguez-Garcia DR, Del Carmen Rondón Guerrero Y, Sardoy MC, Ferrero L, Botto JF, Meneses C, Ariel F, Nadra AD, Petrillo E, Dunand C, Estevez JM** 2022 Apoplastic class III peroxidases PRX62 and PRX69 promote Arabidopsis root hair growth at low temperature. Nat Commun **13:** 1310

**Pedreira J, Herrera MT, Zarra I, Revilla G** 2011 The overexpression of AtPrx37, an apoplastic peroxidase, reduces growth in Arabidopsis. Physiol Plant **141:** 177-187

**Pereira AM, Masiero S, Nobre MS, Costa ML, Solís MT, Testillano PS, Sprunck S, Coimbra S** 2014 Differential expression patterns of arabinogalactan proteins in Arabidopsis thaliana reproductive tissues. J Exp Bot **65:** 5459-5471

**Pitaksaringkarn W, Matsuoka K, Asahina M, Miura K, Sage-Ono K, Ono M, Yokoyama R, Nishitani K, Ishii T, Iwai H, Satoh S** 2014 XTH20 and XTH19 regulated by ANAC071 under auxin flow are involved in cell proliferation in incised Arabidopsis inflorescence stems. Plant J **80:** 604-614

**Shanmugarajah K, Linka N, Gräfe K, Smits SHJ, Weber APM, Zeier J, Schmitt L** 2019 ABCG1 contributes to suberin formation in Arabidopsis thaliana roots. Sci Rep **9:** 11381

**Shigeto J, Itoh Y, Hirao S, Ohira K, Fujita K, Tsutsumi Y** 2015 Simultaneously disrupting AtPrx2, AtPrx25 and AtPrx71 alters lignin content and structure in Arabidopsis stem. J Integr Plant Biol **57:** 349-356

**Su T, Xu J, Li Y, Lei L, Zhao L, Yang H, Feng J, Liu G, Ren D** 2011 Glutathione-indole-3-acetonitrile is required for camalexin biosynthesis in Arabidopsis thaliana. Plant Cell **23:** 364-380

**Xing DH, Lai ZB, Zheng ZY, Vinod KM, Fan BF, Chen ZX** 2008 Stress- and pathogen-induced Arabidopsis WRKY48 is a transcriptional activator that represses plant basal defense. Mol Plant **1:** 459-470

**Wieczorek K, Golecki B, Gerdes L, Heinen P, Szakasits D, Durachko DM, Cosgrove DJ, Kreil DP, Puzio PS, Bohlmann H, Grundler FM** 2006 Expansins are involved in the formation of nematode-induced syncytia in roots of Arabidopsis thaliana. Plant J **48:** 98-112

**Xu Z, Escamilla-Treviño L, Zeng L, Lalgondar M, Bevan D, Winkel B, Mohamed A, Cheng CL, Shih MC, Poulton J, Esen A** 2004 Functional genomic analysis of Arabidopsis thaliana glycoside hydrolase family 1. Plant Mol Biol **55:** 343-367

**Zhang C, He M, Jiang Z, Liu L, Pu J, Zhang W, Wang S, Xu F** 2022 The Xyloglucan Endotransglucosylase/Hydrolase Gene XTH22/TCH4 Regulates Plant Growth by Disrupting the Cell Wall Homeostasis in Arabidopsis under Boron Deficiency. Int J Mol Sci **23**
