# Supplementary material for: Syntaxin of plants71 plays essential roles in plant development and stress response via regulating pH homeostasis
Source: Front Plant Sci. 2023 Jun 5;14:1198353. doi: 10.3389/fpls.2023.1198353 (PMC10277689; doi:10.3389/fpls.2023.1198353)
Supplement: Supplementary Table 3 — Primer information used in this study. [file Table_3.docx]

**Supplementary Table 3. All primer sequences used in this study**

| **name** | **sequences (5‘-3’)** | **experiment** |
| --- | --- | --- |
| *atsyp71-4*-PCR-LP | TAGTGAATTCGGTGTTGGTCC | atsyp71-4 genotyping |
| *atsyp71-4*-PCR-RP | TCATTCCCATCACGAAAACTC | atsyp71-4 genotyping |
| *atsyp71-2*-PCR-LP | ACAAGATCGTTGGTTTCCATG | atsyp71-2 genotyping |
| *atsyp71-2*-PCR-RP | ATTATTTCGTGATCCACGAGG | atsyp71-2 genotyping |
| *atsyp71-3*-PCR-LP | AGGAAAAATGTTTTGGCTTCC | atsyp71-3 genotyping |
| *atsyp71-3*-PCR-RP | AGCACTAGTGCTTTTAGGGCC | atsyp71-3 genotyping |
| *atsyp71-1*-PCR-LP | AGGTGGCCCTAAAAGCACTAG | atsyp71-1 genotyping |
| *atsyp71-1*-PCR-RP | CTATGCACACGAACAACATGG | atsyp71-1 genotyping |
| actin2-qPCR-F | AGAGATTCAGATGCCCAGAAGTCTTGTT | actin2 RT-qPCR |
| actin2-qPCR-R | AACGATTCCTGGACCTGCCTCATCATACTC | actin2 RT-qPCR |
| AtSYP71-qPCR-F | CAAGTACGATGTCGACAAACAG | AtSYP71 RT-qPCR |
| AtSYP71-qPCR-R | CCCTGTTTTTCTCCTTCGTAAC | AtSYP71 RT-qPCR |
| PRX4-qPCR-F | CTTCTCTCATCCGTCTCCATTTC | PRX4 RT-qPCR |
| PRX4-qPCR-R | GCCAATGAATCTCTCTCACTCTC | PRX4 RT-qPCR |
| PRX15-qPCR-F | GAACCTCTTCCCAGGTTTCTAC | PRX15 RT-qPCR |
| PRX15-qPCR-R | CTCATGAGAGAAGCAGCCATAC | PRX15 RT-qPCR |
| PRX25-qPCR-F | GAGTACCAACGGGTCGTAAAG | PRX25 RT-qPCR |
| PRX25-qPCR-R | AGAGTCAAGTGGAGAAGGTAGA | PRX25 RT-qPCR |
| PRX37-qPCR-F | CCAGAGCGACCAAGAGTTATT | PRX37 RT-qPCR |
| PRX37-qPCR-R | CCCTTGACCATCAGCGTATT | PRX37 RT-qPCR |
| PRX49-qPCR-F | CTCACGAGCTTTCTCCTTCTTC | PRX49 RT-qPCR |
| PRX49-qPCR-R | GGGCATGAATGGGCGTAATA | PRX49 RT-qPCR |
| PRX52-qPCR-F | CGAAGCACAACTCACGACTAA | PRX52 RT-qPCR |
| PRX52-qPCR-R | GGAAGAAGAGACGGAGGATAGA | PRX52 RT-qPCR |
| PRX53-qPCR-F | GGCTGTCGGGCTTAATACAA | PRX53 RT-qPCR |
| PRX53-qPCR-R | CCTGTCCCGCTGAAGTTAAATA | PRX53 RT-qPCR |
| PRX54-qPCR-F | CTGGGAGTAGTGGAGAGATTAGA | PRX54 RT-qPCR |
| PRX54-qPCR-R | ACTGGTCCGTCAGATTGTAAC | PRX54 RT-qPCR |
| PRX62-qPCR-F | CGGGACCTAACTCTGAAAGAAC | PRX62 RT-qPCR |
| PRX62-qPCR-R | CAAGAGACAACACCAGGACAT | PRX62 RT-qPCR |
| AT3G03640-qPCR-F | GTCTGCGAGGACAAGGTTAAT | AT3G03640 RT-qPCR |
| AT3G03640-qPCR-R | CCGTCCCATATTGTGTCCATAG | AT3G03640 RT-qPCR |
| BGAL8-qPCR-F | CTGATGCAACTGTGACTTTCAA | BGAL8 RT-qPCR |
| BGAL8-qPCR-R | CTTTGCGGTATTGAAAGCTACA | BGAL8 RT-qPCR |
| SCPL12-qPCR-F | CCCACTTATTGATACGCCTAGT | SCPL12 RT-qPCR |
| SCPL12-qPCR-R | ATTGGAAGAAAACTGTGGATGC | SCPL12 RT-qPCR |
| LTPG1-qPCR-F | CTGATGAATGCAACCAGGATTT | LTPG1 RT-qPCR |
| LTPG1-qPCR-R | TCACAACACTTCTTAGACGGAA | LTPG1 RT-qPCR |
| LTP2-qPCR-F | TAAAAACATGGCCAGTACAACC | LTP2 RT-qPCR |
| LTP2-qPCR-R | ATATTGACTTTGCATGCGCTAG | LTP2 RT-qPCR |
| LTP5-qPCR-F | TTGGTGATTGTGTGCATGTTAG | LTP5 RT-qPCR |
| LTP5-qPCR-R | CCTCGGGTCAAGTAGTTATAGC | LTP5 RT-qPCR |
| ATEXPA1-qPCR-F | ACGGAAACCTATATAGCCAAGG | ATEXPA1 RT-qPCR |
| ATEXPA1-qPCR-R | ACGGAAACCTATATAGCCAAGG | ATEXPA1 RT-qPCR |
| EXPA4-qPCR-F | ACGGAAACCTATATAGCCAAGG | EXPA4 RT-qPCR |
| EXPA4-qPCR-R | ACGGAAACCTATATAGCCAAGG | EXPA4 RT-qPCR |
| AT4G20830-qPCR-F | ACGGAAACCTATATAGCCAAGG | AT4G20830 RT-qPCR |
| AT4G20830-qPCR-R | ACGGAAACCTATATAGCCAAGG | AT4G20830 RT-qPCR |
| AT5G44400-qPCR-F | ACGGAAACCTATATAGCCAAGG | AT5G44400 RT-qPCR |
| AT5G44400-qPCR-R | ACGGAAACCTATATAGCCAAGG | AT5G44400 RT-qPCR |
| AT1G30730-qPCR-F | ACGGAAACCTATATAGCCAAGG | AT1G30730 RT-qPCR |
| AT1G30730-qPCR-R | ACGGAAACCTATATAGCCAAGG | AT1G30730 RT-qPCR |
| XTH20-qPCR-F | ACGGAAACCTATATAGCCAAGG | XTH20 RT-qPCR |
| XTH20-qPCR-R | ACGGAAACCTATATAGCCAAGG | XTH20 RT-qPCR |
| XTH22-qPCR-F | ACGGAAACCTATATAGCCAAGG | XTH22 RT-qPCR |
| XTH22-qPCR-R | ACGGAAACCTATATAGCCAAGG | XTH22 RT-qPCR |
| AT4G01700-qPCR-F | ACGGAAACCTATATAGCCAAGG | AT4G01700 RT-qPCR |
| AT4G01700-qPCR-R | ACGGAAACCTATATAGCCAAGG | AT4G01700 RT-qPCR |
| AT5G18470-qPCR-F | ACGGAAACCTATATAGCCAAGG | AT5G18470 RT-qPCR |
| AT5G18470-qPCR-R | ACGGAAACCTATATAGCCAAGG | AT5G18470 RT-qPCR |
| GSTF6-qPCR-F | ACGGAAACCTATATAGCCAAGG | GSTF6 RT-qPCR |
| GSTF6-qPCR-R | ACGGAAACCTATATAGCCAAGG | GSTF6 RT-qPCR |
| CYP706A1-qPCR-F | ACGGAAACCTATATAGCCAAGG | CYP706A1 RT-qPCR |
| CYP706A1-qPCR-R | ACGGAAACCTATATAGCCAAGG | CYP706A1 RT-qPCR |
| ABCG1-qPCR-F | ATTGCTCAGAGTGGTAGTATCG | ABCG1 RT-qPCR |
| ABCG1-qPCR-R | TGACAAGAAGATTAACCGGTCA | ABCG1 RT-qPCR |
| PP2-B13-qPCR-F | GCTTACGGACTAGACCTAGTTC | PP2-B13 RT-qPCR |
| PP2-B13-qPCR-R | CCGTAAAATAGCCGCTTCATTT | PP2-B13 RT-qPCR |
| MYB15-qPCR-F | GCCTGATATTAAACGTGGCAAT | MYB15 RT-qPCR |
| MYB15-qPCR-R | CGAGTCTCTTCTTCAAGTGAGT | MYB15 RT-qPCR |
| HRS1-qPCR-F | TCTCAAACGCAACATTTCGTAG | HRS1 RT-qPCR |
| HRS1-qPCR-R | ATCCTTCGTCCACAATAATCGA | HRS1 RT-qPCR |
| JAL22-qPCR-F | GGAAAGGAAGATGCAGCTATTG | JAL22 RT-qPCR |
| JAL22-qPCR-R | CCATCATCCCATGTAACTCCTT | JAL22 RT-qPCR |
| AT3G16530-qPCR-F | TTACTGGGTTCAGACAAGAGTC | AT3G16530 RT-qPCR |
| AT3G16530-qPCR-R | CGAGTGTAACCGTAATCGTAGA | AT3G16530 RT-qPCR |
| WRKY18-qPCR-F | GAAAAACTGAGAACAGCTCCAG | WRKY18 RT-qPCR |
| WRKY18-qPCR-R | TGTCCGTATTTCCTCCATTGAA | WRKY18 RT-qPCR |
| WRKY46-qPCR-F | GTTTAGCAGTGAAGCAAGTTCA | WRKY46 RT-qPCR |
| WRKY46-qPCR-R | CTTCCGATTGCTCTGTAACATG | WRKY46 RT-qPCR |
| WRKY48-qPCR-F | TCCCCAATAACAACAACAACAC | WRKY48 RT-qPCR |
| WRKY48-qPCR-R | TTCTTCTTTGCCTTCAACTGTG | WRKY48 RT-qPCR |
| WRKY51-qPCR-F | CGCAACAACGTTGAGTAAAAAG | WRKY51 RT-qPCR |
| WRKY51-qPCR-R | TCACATCAATCTTCGATCTCGT | WRKY51 RT-qPCR |
| LAC12-qPCR-F | GCTCGACCAAAGAGACTGTTGTAG | LAC12 RT-qPCR |
| LAC12-qPCR-R | GTTTGGCCTGGACCTAACATAAGG | LAC12 RT-qPCR |
| AGP1-qPCR-F | CTTCCGCAGTGGCTCAATCTC | AGP1 RT-qPCR |
| AGP1-qPCR-R | AATCAGCTAGAGGCGGCGATG | AGP1 RT-qPCR |
| MED37C-qPCR-F | GTGATCCCTCTGTTCAAGCGG | MED37C RT-qPCR |
| MED37C-qPCR-R | CCATCGACGAGATTTCCTCAGCAG | MED37C RT-qPCR |
| AtSYP71-AD/BK-F | ATGACTGTGATCGATATTCTG | Yeast Two Hybrid Assay |
| AtSYP71-AD/BK-R | CTTCAGTACATTGTATAAGTATG | Yeast Two Hybrid Assay |
| AtSYP131-AD/BK-F | ATGAACGACCTCTTAAAGGG | Yeast Two Hybrid Assay |
| AtSYP131-AD/BK-R | AGGCACCATTTTTCTGTGTC | Yeast Two Hybrid Assay |
| AtSEC20-AD/BK-F | ATGGATGAGGTTGTTGTTG | Yeast Two Hybrid Assay |
| AtSEC20-AD/BK-R | TAGTTCATCGTGCATGCG | Yeast Two Hybrid Assay |
| AtVTI11-AD/BK-F | ATGAGTGACGTGTTTGATGG | Yeast Two Hybrid Assay |
| AtVTI11-AD/BK-R | CTTGGTGAGTTTGAAGTAC | Yeast Two Hybrid Assay |
